# Gene duplication and evolution in recurring polyploidization–diploidization cycles in plants

**DOI:** 10.1186/s13059-019-1650-2

**Published:** 2019-02-21

**Authors:** Xin Qiao, Qionghou Li, Hao Yin, Kaijie Qi, Leiting Li, Runze Wang, Shaoling Zhang, Andrew H. Paterson

**Affiliations:** 1 Centre of Pear Engineering Technology Research, State Key Laboratory of Crop Genetics and Germplasm Enhancement, Nanjing Agricultural University, Nanjing, 210095 China; 2 Plant Genome Mapping Laboratory, University of Georgia, Athens, GA 30605 USA

**Keywords:** Gene duplication, Evolution, Polyploidization, Gene conversion, Plant

## Abstract

**Background:**

The sharp increase of plant genome and transcriptome data provide valuable resources to investigate evolutionary consequences of gene duplication in a range of taxa, and unravel common principles underlying duplicate gene retention.

**Results:**

We survey 141 sequenced plant genomes to elucidate consequences of gene and genome duplication, processes central to the evolution of biodiversity. We develop a pipeline named *DupGen_finder* to identify different modes of gene duplication in plants. Genes derived from whole-genome, tandem, proximal, transposed, or dispersed duplication differ in abundance, selection pressure, expression divergence, and gene conversion rate among genomes. The number of WGD-derived duplicate genes decreases exponentially with increasing age of duplication events—transposed duplication- and dispersed duplication-derived genes declined in parallel. In contrast, the frequency of tandem and proximal duplications showed no significant decrease over time, providing a continuous supply of variants available for adaptation to continuously changing environments. Moreover, tandem and proximal duplicates experienced stronger selective pressure than genes formed by other modes and evolved toward biased functional roles involved in plant self-defense. The rate of gene conversion among WGD-derived gene pairs declined over time, peaking shortly after polyploidization. To provide a platform for accessing duplicated gene pairs in different plants, we constructed the Plant Duplicate Gene Database.

**Conclusions:**

We identify a comprehensive landscape of different modes of gene duplication across the plant kingdom by comparing 141 genomes, which provides a solid foundation for further investigation of the dynamic evolution of duplicate genes.

**Electronic supplementary material:**

The online version of this article (10.1186/s13059-019-1650-2) contains supplementary material, which is available to authorized users.

## Background

The finding that the first fully sequenced eukaryote genome, that of the budding yeast (*Saccharomyces cerevisiae*) [1], had experienced whole-genome duplication (WGD, or defined as polyploidization) [2] invigorated research into this evolutionary mechanism of central importance. The otherwise compact ciliate (*Paramecium tetraurelia*) genome (72 Mb) has nonetheless retained a high number of gene sets (40,000) after at least three successive whole-genome duplications [3–5]. The ancestral vertebrate is thought to have undergone two rounds of ancient WGD (defined as 1R and 2R) at least ~ 450 million years ago (Mya) [6–8]—about 20–30% of human genes are thought to be paralogs produced by these two WGDs, and these “ohnologs” have a strong association with human disease [7, 9]. Additional WGDs occurred in the common ancestor of teleost fish (3R, ~ 320 Mya) [10, 11] and salmonids including the rainbow trout (*Oncorhynchus mykiss*) and Atlantic salmon (*Salmo salar*) (4R) dated to ~ 80 Mya [12, 13]. The most recent genome duplication currently known in vertebrates has been uncovered in the common carp (*Cyprinus carpio*) (4R, ~ 8.2 Mya) [14, 15].

In contrast with fungi and animals, the most frequent occurrence of paleo-polyploidization has been detected in angiosperms, flowering plants. It has been suggested that one to two genome duplications preceded angiosperm diversification [16], and only one angiosperm is known that did not experience additional WGDs, *Amborella trichopoda* [17]. *Arabidopsis thaliana*, chosen to be the first fully sequenced angiosperm in part due to its apparent genomic simplicity, is, ironically, a member of the Brassicaceae family that is as yet unmatched in its propensity for genome duplication—*Brassica napus* has experienced an aggregate 72× multiplication, in five events (3 × 2 × 2 × 3 × 2) at times ranging from > 100 million to ~ 10,000 years ago [18]. A WGD series of rho (*ρ*)–sigma (*σ*)–tau (*τ*) in Poaceae [19] echoes the now-classic alpha (*α*)–beta (*β*)–gamma (*γ*) series in Brassicaceae [20]. While most plant paleopolyploidies are duplications, several are triplications [21–23] and at least one is a penta-plication [24].

Whole-genome duplication is thought to have contributed much to the evolution of morphological and physiological diversity [25, 26]. However, WGD is often followed by loss of most duplicated genes over a few million years [27] and is episodic [19, 20]. Successive WGD events are often separated by tens of millions of years, failing to provide a continuous supply of variants available for adaptation to continuously changing environments. Diploidization is thought to occur “quickly” (i.e., in the first few million years, [27]) following WGD to return to disomic inheritance, by genome modifications including chromosomal rearrangement, gene loss, gene conversion, subgenome dominance, and expression divergence between duplicate copies [28–30]. The tiny genome (82 Mb) of bladderwort (*Utricularia gibba*) which accommodates a typical number of genes for a plant but purges almost all intergenic DNA and repeat sequence exemplifies the extreme genome reduction or fractionation after multiple rounds of WGD [31].

With a diploidized state restored soon after genome duplication, what is the raw material for adaptation in taxa that have abstained from genome duplication for long time periods? Various types of single-gene duplication occur more or less continuously and have been implicated in key environmental adaptations [32, 33], but yield genes with short half-lives [27]. De novo gene evolution, for example as a result of transposable element activities [34], may often form fragmentary products of uncertain function [35]. In addition to whole-genome duplication, other modes of gene duplication are collectively deemed single-gene duplications [36–38]. Single genes can move, or be copied, from the original chromosomal position to a new position by various ways [39–41]. Tandem duplicates are closely adjacent to each other in the same chromosome, a phenomenon which is speculated to occur through unequal crossing over [36]. Proximal duplication (PD) generates gene copies that are near each other but separated by several genes (10 or fewer genes), possibly through localized transposon activities [42] or originating from ancient tandem duplicates interrupted by other genes [39]. It has been revealed that neighboring genes tend to be co-regulated, especially tandem duplicates [43], and neighboring gene pairs still show interchromosomal colocalization after their separation [44]. Moreover, tandem duplicates have been commonly found to be important for plant adaptation to rapidly changing environments [45]. The transposed duplication (TRD) generates a gene pair comprised of an ancestral and a novel locus and is presumed to arise through distantly transposed duplications occurred by DNA-based or RNA-based mechanisms [38, 46]. Dispersed duplication (DSD) happens through unpredictable and random patterns by mechanisms that remain unclear, generating two gene copies that are neither neighboring nor colinear [47]. The dispersed duplicates are prevalent in different plant genomes [48].

Herein, we exploited a pipeline incorporating syntenic and phylogenomic approaches to identify the different modes of gene duplication in 141 sequenced plant genomes. Duplicated genes were classified into five types, including whole-genome duplication, tandem duplication (TD), proximal duplication, transposed duplication, and dispersed duplication. Integrated large-scale genome and transcriptome datasets were used to investigate selection pressures, expression divergence, and gene conversion underlying duplicate gene evolution. In addition, construction of gene families using all genes from 141 plant genomes suggested 232 families most widely preserved across the plant kingdom. The results of this study lay a substantial foundation for further investigating the contributions of gene duplication to gene regulatory network evolution, epigenetic variation, morphological complexity, and adaptive evolution in plants.

## Results

### The landscape of gene duplication in the plant kingdom

**Fig. 1:**
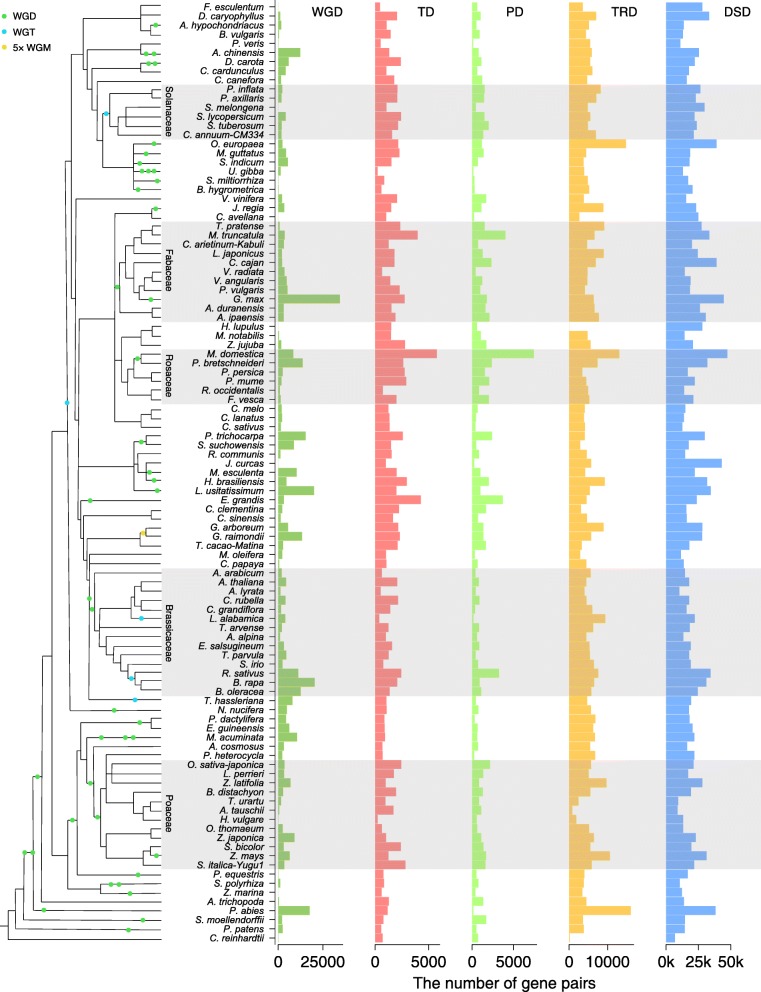
The number of gene pairs derived from different modes of duplication in representative plant genomes. WGD whole-genome duplication, TD tandem duplication, PD proximal duplication, TRD transposed duplication, DSD dispersed duplication. A schematic diagram of phylogeny of different plant species [231–233] and the WGDs occurred in different branches were labeled. Branch length is not directly proportional to time scale. WGT whole-genome triplication, WGM whole-genome multiplication

In 141 sequenced plant genomes, we identified duplicated genes using *DupGen_finder* (freely available at https://github.com/qiao-xin/DupGen_finder) and classified them into one of the five categories (Additional file 1: Figure S1 and Additional file 2), being derived from WGD, TD, PD, TRD, and DSD. The number of duplicate gene pairs for each category in each taxon was determined (Fig. 1 and Additional file 3). The higher percentages of WGD-derived gene pairs were detected in plants experiencing more recent WGDs such as soybean (*Glycine max*, ~ 13 Mya) and flax (*Linum usitatissimum*, 3.7~6.8 Mya). Interestingly, the highest frequency of whole-genome triplication (WGT) occurred in plants belonging to Brassicaceae such as cabbage (*Brassica oleracea*), radish (*Raphanus sativus*), and *Leavenworthia alabamica*. In addition, the occurrence of genome duplication is frequent in some individual plants such as kiwifruit (*Actinidia chinensis*, two rounds of WGD), carrot (*Daucus carota*, a WGT (Dc-*β*) and a WGD (Dc-*α*)), and banana (*Musa acuminata*, three rounds of WGD). Larger percentages of WGD-derived gene pairs are still maintained in the aforementioned species although genome fractionation occurred quickly after genome duplication. To provide a platform for accessing and searching duplicated gene in 141 sequenced plants, we constructed a public database named Plant Duplicate Gene Database (PlantDGD, freely available at http://pdgd.njau.edu.cn:8080).

### Identifying *K*_s_ peaks corresponding to genome duplication events of different ages in each species

The most recent and more ancient genome duplication events that affect each of the taxa were identified (Additional file 4). To identify the most recent and more ancient *K*_s_ peaks (or WGDs) in each species, we estimated the mean *K*_s_ values for the gene pairs contained in each syntenic block within a species, and in addition, the *K*_s_ distribution was fitted using Gaussian mixture models (GMM) (the code is freely available at https://github.com/qiao-xin/Scripts_for_GB).

**Fig. 2:**
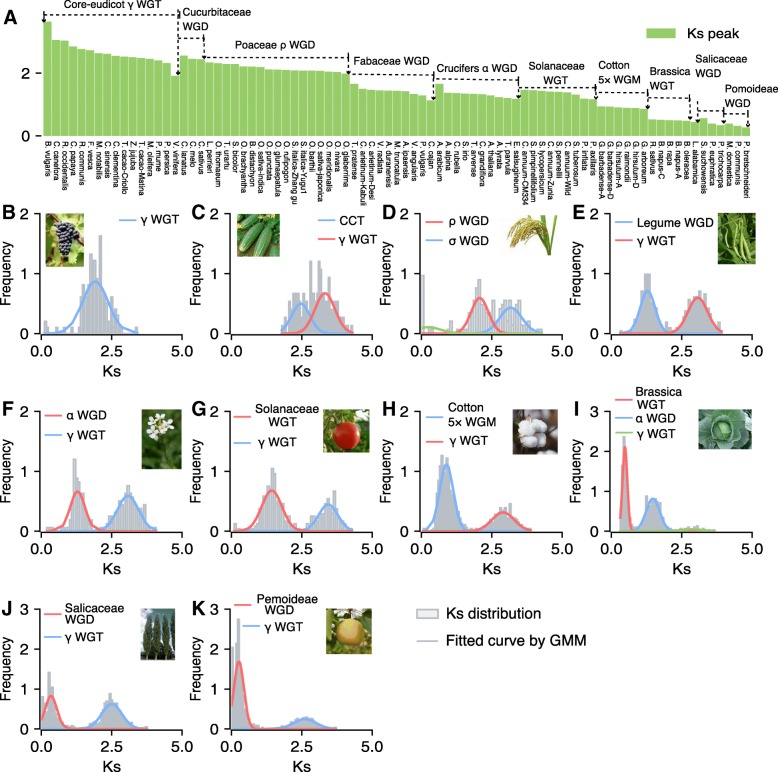
Lineage-specific genome duplication events. **a** The genome duplication events identified in different lineages. A range of *K*_s_ values for estimates of individual genome duplication events from different taxa. The *K*_s_ distribution in each species was fitted using Gaussian mixture models (GMM). The *K*_s_ peaks corresponding to core eudicot *γ* WGT, cucurbit-common tetraploidization (CCT), Poaceae *ρ* WGD, Fabaceae common WGD, Brassicaceae *α* WGD, Solanaceae WGT, cotton 5× WGM, Brassica WGT, Salicaceae WGD, and Pomoideae WGD were respectively detected in *Vitis vinifera* (**b**), *Cucumis sativus* (**c**), *Oryza sativa* (**d**), *Phaseolus vulgaris* (**e**), *Arabidopsis thaliana* (**f**), *Solanum lycopersicum* (**g**), *Gossypium raimondii* (**h**), *Brassica oleracea* (**i**), *Populus trichocarpa* (**j**), and *Pyrus bretschneideri* (**k**)

Ranges of *K*_s_ values for estimates of individual genome duplication events (e.g., *γ* WGT in core eudicots) from different taxa reflect substantial divergence in evolutionary rates (clock-like rates, substitutions/synonymous site/year) in specific lineages (Fig. 2 and Additional file 4). There are 16 species which have not been influenced by recent genome duplication event but share the core eudicot *γ* WGT events. The *K*_s_ peaks corresponding to the *γ* WGT from these 16 taxa range from 1.91 to 3.64 (Fig. 2a). For example, we detected strong signal of *γ* WGT in grape (*Vitis vinifera*) (Fig. 2b). The *K*_s_ values corresponding to the cucurbit-common tetraploidization (CCT) range from 2.44 to 2.56. The *K*_s_ values corresponding to the Poaceae *ρ* WGD range from 1.98 to 2.34. For example, two *K*_s_ peaks corresponding to *ρ* WGD and *σ*/*τ* WGD were detected in rice (*Oryza sativa*) (Fig. 2d). The *K*_s_ values corresponding to the Fabaceae common WGD range from 1.13 to 1.66. The *K*_s_ values corresponding to the Brassicaceae *α*/*β* WGD range from 1.18 to 1.66. For example, two *K*_s_ peaks corresponding to *α*/*β* WGD and *γ* WGT were fitted by a GMM method in *Arabidopsis* (Fig. 2f). The *K*_s_ values corresponding to the Solanaceae common WGT range from 1.17 to 1.46. The *K*_s_ values corresponding to the cotton 5× WGM (whole-genome multiplication) range from 0.86 to 0.93. The *K*_s_ values corresponding to the Brassica common WGT range from 0.48 to 0.52. For example, three *K*_s_ peaks corresponding to Brassica WGT, *α*/*β* WGD, and *γ* WGT respectively, were fitted in *Brassica oleracea* (Fig. 2i). The *K*_s_ values corresponding to the Salicaceae common WGD range from 0.34 to 0.56. The *K*_s_ values corresponding to the Pomoideae WGD range from 0.27 to 0.39.

### Dynamic changes in abundance of duplicated genes over time

**Fig. 3:**
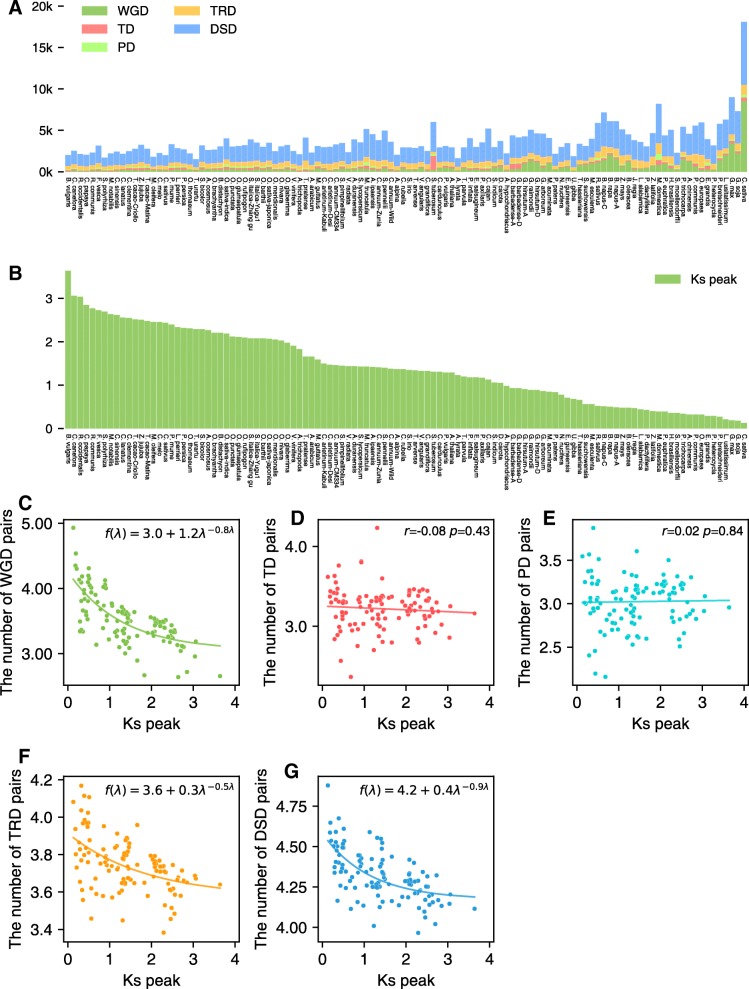
Changes in abundance of different modes of duplicated gene pairs over time. **a** The distribution of number of gene pairs derived from different modes of duplication in 141 plant genomes. Genomes with abnormal *K*_s_ peaks because fragmented assembly hindered the identification of large syntenic blocks were not included. **b** The fitted *K*_s_ peak corresponding to the most recent WGD for each species. **c**–**g** The relationship between the log10-transformed number of different types of gene pairs and *K*_s_ peak of WGD genes from different taxa, excluding those taxa with abnormal *K*_s_ peaks due to fragmented assembly. **c** WGD-pairs. **d** TD-pairs: transposed gene pairs. **e** PD-pairs: proximal gene pairs. **f** TRD-pairs: transposed gene pairs. **g** DSD-pairs: dispersed gene pairs. Exponential fit and linear regression analysis were performed. The exponential equation was annotated in subplots **c**, **f**, and **g**; Pearson correlation coefficient (*r*) was annotated in subplots **d** and **e**

The most recent *K*_s_ peaks were used to determine the order in which the taxa are shown in Fig. 3a (types of gene duplications). Genomes with abnormal *K*_s_ peaks were not included in Fig. 3a because fragmented assembly hindered the identification of large syntenic blocks. We detected whole-genome duplication in all plant genomes investigated except for several with highly fragmented assemblies such as Hop (*Humulus lupulus*) and European hazelnut (*Corylus avellana*). The *K*_s_ values for duplication events show a steady decline with decreasing antiquity (Fig. 3b), as expected.

Linear regression between the number of each type of duplicated gene pair and the *K*_s_ peaks from different taxa showed that the number of gene pairs derived from WGD generally declines with increasing antiquity of duplication events (*r* = − 0.45, *P* < 0.001, Additional file 1: Figure S2A), although again with substantial fluctuation among taxa (Fig. 3a). Paralleling the decline in WGD-derived gene pairs with increasing antiquity is decreases in TRD-derived (*r* = − 0.50, *P* < 0.001) or DSD-derived gene pairs (*r* = − 0.57, *P* < 0.001) (Additional file 1: Figure S2D and E). Tandem and proximal duplicate pairs show a nominal (nonsignificant) decrease (*r* = − 0.11, *P* = 0.25 and *r* = − 0.10, *P* = 0.28) (Additional file 1: Figure S2B and C).

Further, the absolute number of duplicate gene pairs for each category in each taxon was converted to log10-transformed number to mitigate the effect of genome size and total gene number variation among taxa. Linear regression between the log10-transformed number of each type of duplicated gene pair and the *K*_s_ peaks from different taxa strongly supported that the number of duplicated gene pairs derived from WGD (*r* = − 0.70, *P* < 0.001), TRD (*r* = − 0.49, *P* < 0.001), and DSD (*r* = − 0.61, *P* < 0.001) significantly declines with increasing antiquity of duplication events (Additional file 1: Figure S3A, D and E). However, the number of tandem and proximal duplicates showed no significant decrease over time (*r* = − 0.08, *P* = 0.43 and *r* = 0.02, *P* = 0.84) and may provide a continuous supply of genes potentially useful for plant adaptation. Moreover, the exponential fit was performed between log10-transformed numbers (*y* axis) and *K*_s_ peaks (*x* axis). The number of WGD-derived pairs decreases exponentially with increasing antiquity of duplication events (Fig. 3c). The chi-squared goodness of fit test supports this observation (or null hypothesis) (*χ*2 = 2.33, *P* = 1.0). Exponential decrease of number of duplicated genes over time was also found in TRD- and DSD-derived duplicate genes (*χ*2 = 0.47, *P* = 1.0 and *χ*2 = 0.37, *P* = 1.0) (Fig. 3f, g). Significant exponential decay was not found in TD- and PD-derived duplicate genes (Fig. 3d, e).

To investigate whether results of the aforementioned linear regression analyses have bias due to some individual genome duplication events being shared among different taxa, we undertook new analyses using only one that sampled each of the most recent genome duplication events (Fig. 2, noting that ancient events were unavoidably shared across species). The results from this new analysis supported that the number of duplicated gene pairs derived from WGD (*r* = − 0.39, *P* < 0.05), TRD (*r* = − 0.46, *P* < 0.001), and DSD (*r* = − 0.56, *P* < 0.001) declines significantly with increasing antiquity of duplication events (Additional file 1: Figure S4A, D and E). The number of tandem and proximal duplicates showed no significant decrease over time (*r* = − 0.22, *P* = 0.17 and *r* = − 0.23, *P* = 0.16) (Additional file 1**:** Figure S4B and C). Linear regression analysis using the log10-transformed number of each type of duplicated gene pair also supported our prior observation (Additional file 1: Figure S5).

### Evolutionary forces inferred to affect duplicated genes

**Fig. 4:**
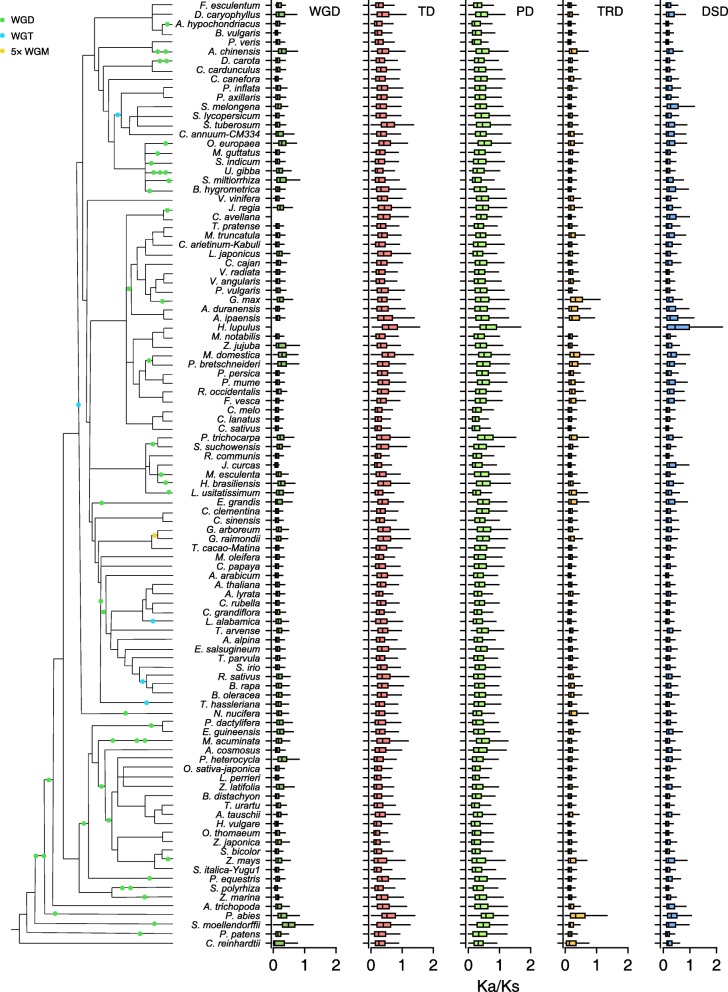
The *K*_a_/*K*_s_ ratio distributions of gene pairs derived from different modes of duplication in representative plant genomes. WGD whole-genome duplication, TD tandem duplication, PD proximal duplication, TRD transposed duplication, DSD dispersed duplication

The *K*_a_ (number of substitutions per nonsynonymous site), *K*_s_ (number of substitutions per synonymous site), and *K*_a_/*K*_s_ values were estimated for gene pairs generated by different modes of duplication. We compared the *K*_a_, *K*_s_, and *K*_a_/*K*_s_ distributions across 141 plants (Fig. 4 and Additional file 1: Figure S6 and S7). The *K*_a_/*K*_s_ ratios among different modes of gene duplications showed a striking trend, with tandem and proximal duplications having qualitatively higher *K*_a_/*K*_s_ ratios than other modes. The TD- and PD-derived gene pairs have relatively smaller *K*_s_ values (Additional file 1: Figure S7). This finding suggests that tandem and proximal duplications of younger age that have been preserved have experienced more rapid sequence divergence than other gene classes, although concerted evolution may also preserve homogeneity of TD or PD genes to a greater degree than genes that are not located near one another. In contrast, WGD genes are more conserved with smaller *K*_a_/*K*_s_ ratios.

We further explored the roles of purifying selection (*K*_a_/*K*_s_ < 1) and positive selection (*K*_a_/*K*_s_ > 1) in the evolution of duplicated genes in seven model plants, including *Arabidopsis thaliana* (eudicots), *Oryza sativa* (monocots), *Amborella trichopoda* (angiosperm, Amborellales), *Picea abies* (Norway spruce, gymnosperms), *Selaginella moellendorffii* (Lycophytes), *Physcomitrella patens* (Bryophytes), and *Chlamydomonas reinhardtii* (Chlorophytes). The majority of duplicated genes evolve under purifying selection (*K*_a_/*K*_s_ < 1) **(**Additional file 1: Table S1 and Figure S8-S14). In *Arabidopsis*, 100% WGD-, 96.5% TD-, 94.9% PD-, 99.7% TRD-, and 99.3% DSD-derived duplicate genes experienced purifying selection, while only 0.0–5.1% of duplicated genes show evidence of positive selection (Additional file 1: Figure S8). Likewise, evidence of purifying selection is found for 98.3–99.7% of duplicated genes in *O. sativa*, 91.9–97.2% in *A. trichopoda*, 86.2–98.8% in *P. abies*, 91.8–98.3% in *S. moellendorffii*, 91.4–99.7% in *P. patens*, and 95.7–98.7% in *C. reinhardtii*. Consistent with our earlier observation, tandem and proximal duplicates experienced stronger positive selection than other modes (Fig. 4 and Additional file 1: Table S1), reflected by the high percentages of gene pairs showing *K*_a_/*K*_s_ > 1 in *Arabidopsis* (PD (5.1%) > TD (3.3%) > DSD (0.6%) > TRD (0.3%) > WGD (0.0%)) and other model plants. This finding suggests that tandem and proximal duplication is an important source of genetic material for evolving new functions.

**Fig. 5:**
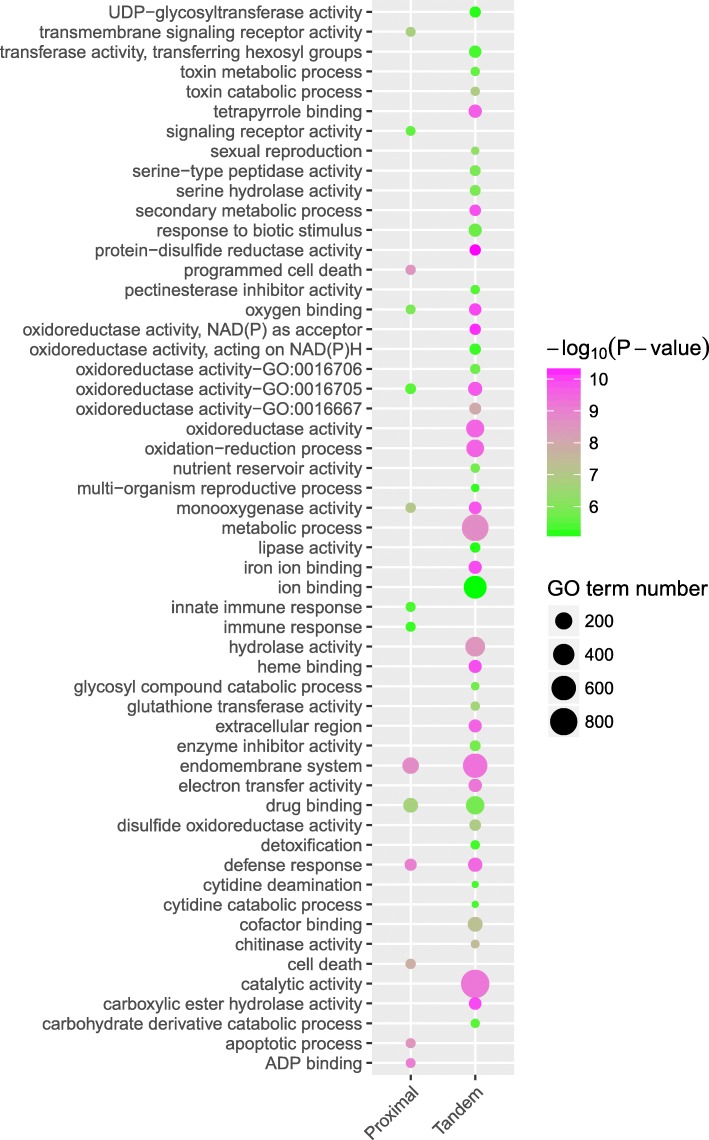
Functional enrichment analysis of tandem and proximal duplicates in *Arabidopsis*. The enriched GO terms with corrected *P* value < 0.01 are presented. The color of circle represents the statistical significance of enriched GO terms. The size of the circles represents the number of occurrences of a GO term

Does stronger selective pressure drive the evolution of tandem and proximal duplicates toward specific biological functions? To answer this question, we performed GO enrichment analysis to investigate the functional roles of tandem and proximal genes in the model plant *A. thaliana*, given its high-quality genome annotation and extensive functional analysis. Tandem and proximal duplicates exhibited divergent functional roles although they shared several enriched GO terms involved in defense response, drug binding, endomembrane system, monooxygenase activity, oxidoreductase activity, and oxygen binding, which are critical for plant self-defense and adaptation (Additional file 5). In particular, proximal duplicates are enriched in GO terms involved in apoptotic processes, cell death, programmed cell death, immune response, and signaling receptor activity. Tandem duplicates are enriched in GO terms involved in “binding,” such as tetrapyrrole binding, iron ion binding, heme binding, and cofactor binding, and “activity” such as transferase activity, hydrolase activity, electron transfer activity, and catalytic activity (Fig. 5).

### Expression divergence between duplicated genes

**Fig. 6:**
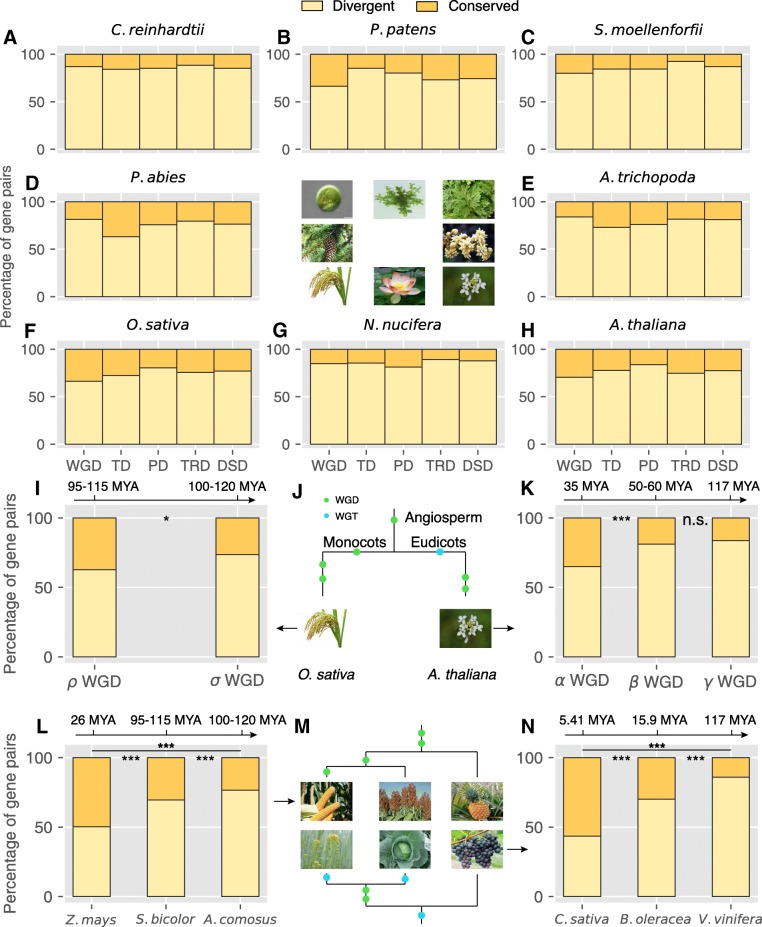
Expression divergence between duplicate genes derived from WGD, tandem (TD), proximal (PD), transposed (TRD), and dispersed (DSD) duplication in **a** *C. reinhardtii*, **b** *P. patens*, **c** *S. moellendorffii*, **d** *P. abies*, **e** *A. trichopoda*, **f** *O. sativa*, **g** *N. nucifera*, and **h** *A. thaliana*. The proportion of gene pairs conserved and divergent in expression, respectively, was indicated by different colors. **i**–**k** The expression divergence between duplicate genes derived from genome duplication events of different ages in model plants *A. thaliana* (eudicot) and *O. sativa* (monocot). **l**–**n** Expression divergence between duplicate genes in eudicot and monocot plants: **l** *Zea mays*, *Sorghum bicolor*, and *Ananas comosus*; **n** *Camelina sativa*, *Brassica oleracea*, and *Vitis vinifera*. **j**, **m** The phylogeny of different species and genome duplication or triplication events occurring in different branches were labeled. Significant differences (Fisher’s exact test): *P < 0.05, **P < 0.01, ***P < 0.001, ^n.s.^*P* > 0.05

Large-scale RNA-seq data from different tissues, development stages, and treatments are available for a range of plant taxa (Additional file 6). Here, we investigated patterns of expression divergence between duplicated genes in eight model plants for WGD, tandem, proximal, transposed, and dispersed gene pairs. Log10-transformed TPM (transcripts per million) values were used as a proxy for expression levels. For duplicated pairs in which both gene copies are expressed in at least one tissue or condition, Pearson’s correlation coefficient (*r*) was calculated between the expression profiles of the two genes, also calculating *r* for 10,000 randomly selected gene pairs for each species. The 95% quantile in the *r* value distribution for random gene pairs was taken as the significance threshold for determining that two gene copies of a duplicated pair have diverged in expression (Additional file 1: Figures S15 and S16). The results showed diverged expression profiles (Fig. 6a–h) for 87%, 66%, 80%, 81%, 84%, 66%, 85%, and 71% of WGD-derived gene pairs in *C. reinhardtii* (Chlorophytes), *P. patens* (Bryophytes), *S. moellendorffii* (Lycophytes), *P. abies* (Norway spruce, gymnosperms), *A. trichopoda* (angiosperm, Amborellales), *O. sativa* (monocots), *N. nucifera* (eudicots, Proteales), and *A. thaliana* (eudicots), respectively. Similarly, 63–85% TD-, 76–85% PD-, 73–92% TRD-, and 74–88% DSD-derived pairs showed expression divergence.

Furthermore, we investigated expression divergence between duplicated genes after genome duplication or triplication events of different ages in strategically chosen monocots and eudicots. Grasses share sigma WGD (*σ*, 100~120 Mya) and tau WGD (*τ*, 110~135 Mya) with *A. comosus* (not including the angiosperm-wide event and beyond) [49]. After divergence from the lineage of *A. comosus*, the common ancestor of grasses including *S. bicolor*, *O. sativa*, and *Z. mays* experienced rho WGD (*ρ*, 95~115 Mya) [50]. In addition, *Z. mays* experienced an additional species-specific event (mWGD, ~ 26 Mya) [50]. Brassicaceae share core eudicot gamma WGT events (*γ*, ~ 117 Mya) with *V. vinifera* [23]. After divergence with *V. vinifera*, the common ancestor of Brassicaceae including *Arabidopsis*, *B. oleracea* and *C. sativa* experienced alpha WGD (*α*, ~ 35 Mya) [21] and beta WGD (*β*, 50~60 Mya) [16, 51]. Following Brassicaceae diversification, *B. oleracea* and *C. sativa* independently experienced species-specific genome triplication events, at ~ 15.9 Mya [21] and ~ 5.41 Mya [52] respectively.

Eudicots *C. sativa*, *B. oleracea* (cabbage), and *V. vinifera* (grape) have been influenced by three different ages of whole-genome triplication, estimated to have occurred at ~ 5.41 [52], ~ 15.9 [21], and ~ 117 [23] Mya respectively. The proportion of WGD-pairs with divergent expression in these three plants increases with the time after duplication from 43 to 86% (*P* < 0.001, Fisher’s exact test) (Fig. 6n), with > 50% of WGD-pairs still undifferentiated in expression ~ 5.41 My after duplication. Monocots *Z. mays* (maize), *S. bicolor* (sorghum), and *A. comosus* (pineapple) also offer stratified ages of whole-genome duplication, at ~ 26 [50], 95~115 [50], and 100~120 [49] Mya respectively. The proportion of WGD-pairs with divergent expression in these three plants increases from 50 to 77% (*P* < 0.001, Fisher’s exact test) (Fig. 6l), with 50% of WGD-pairs showing undifferentiated expression after ~ 26 My.

Moreover, the model plant *Arabidopsis* alone provided three genome duplications: alpha (*α*), beta (*β*), and gamma (*γ*). The proportion of gene pairs with divergent expression from these three WGD events increases from 65 to 84% (*P* < 0.001, Fisher’s exact test) (Fig. 6k). The cereal crop rice provided two rounds of genome duplication, rho (*ρ*) and sigma (*σ*). The proportion of gene pairs with divergent expression from these two WGD events increases from 63 to 74% (*P* < 0.05, Fisher’s exact test) (Fig. 6i). These results indicated that WGD-derived gene pairs show gradually increasing expression divergence with age.

### The rate of gene conversion between WGD-derived paralogs declined over time

**Fig. 7:**
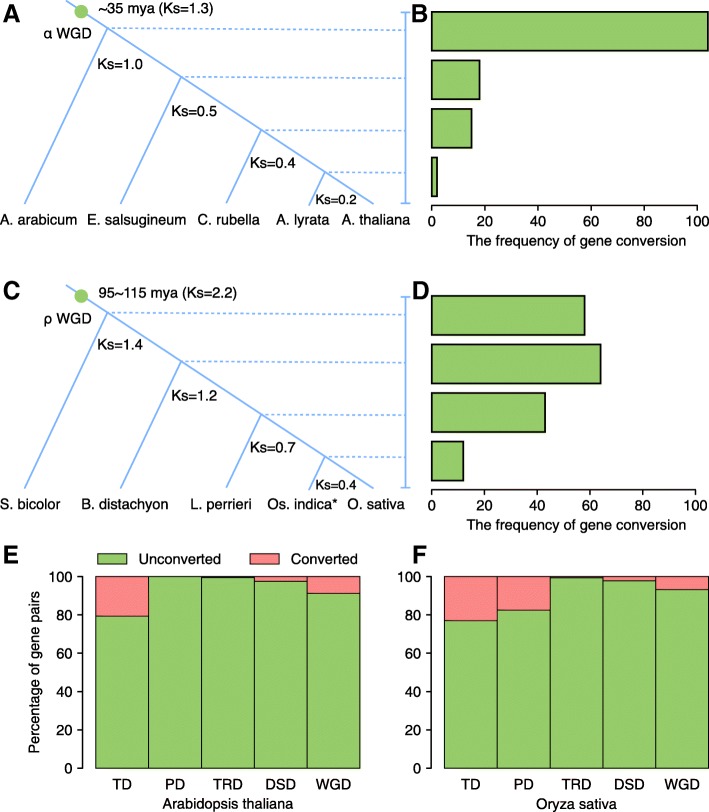
Factors affecting gene conversion rates in plants. **a**, **b** Gene conversion rates after divergence between the *A. thaliana* lineage and those of *Aethionema arabicum*, *Eutrema salsugineum*, *Capsella rubella*, or *A. lyrata*, respectively. **c**, **d** Gene conversion rates after divergence between the *O. sativa* L. (ssp. japonica) lineage and those of *Sorghum bicolor*, *Brachypodium distachyon*, *Leersia perrieri*, or *O. sativa* L. (ssp. indica), respectively. **e**, **f** The frequency of gene conversion events for different modes of duplicated gene pairs in model plants *Arabidopsis* (**e**) and rice (**f**). *O. sativa L. (ssp. indica). WGD whole-genome duplication, TD tandem duplication, PD proximal duplication, TRD transposed duplication, DSD dispersed duplication

We investigated the gene conversion rates of duplicated genes derived from Brassicaceae *α* WGD and Poaceae *ρ* WGD over evolutionary time (Fig. 7). Firstly, high-confidence *α* WGD-derived gene pairs from *A. thaliana* were retrieved from a previous report [20]. The gene conversion rates after divergence between the *A. thaliana* lineage and those of *Aethionema arabicum*, *Eutrema salsugineum*, *Capsella rubella*, or *Arabidopsis lyrata* were examined respectively. *K*_s_ was used as a proxy for evolutionary time. Brassicaceae *α* WGD has been dated to ~ 35 Mya [21]. In this study, the average of a range of *K*_s_ values for estimates of the Brassicaceae *α* WGD event from different Brassicaceae plants is approximately 1.3. To estimate the time of speciation, we calculated the mean *K*_s_ values for the gene pairs contained in each syntenic block between the *Arabidopsis* lineage and each outgroup species, and further, the *K*_s_ distribution was fitted using Gaussian mixture models (GMM) (Additional file 1: Figures S17 and S18). The divergence between the *Arabidopsis* lineage and *A. arabicum* occurred shortly after *α* WGD and dated to *K*_s_ = 1.0. The divergences between the *Arabidopsis* lineage and *E. salsugineum*, *C. rubella*, and *A. lyrata* were respectively dated to 0.5, 0.4, and 0.2. The number of gene conversion events among *α* WGD-derived duplicated gene pairs is 104 after the divergence of *Arabidopsis* and *A. arabicum*, over 50-fold higher than the number after the divergence of *Arabidopsis* and *A. lyrata* (Fig. 7a, b). This result indicated that gene conversion was extensive shortly after polyploidization and declined over time, a result that has been strongly supported by independent evidence [53]. Moreover, the gene conversion rates after divergence between *Oryza sativa* L. (ssp. japonica) lineage and those of *Sorghum bicolor*, *Brachypodium distachyon*, *Leersia perrieri*, or *Oryza sativa* L. (ssp. indica) were examined, using high-confidence Poaceae *ρ* WGD-derived gene pairs from *O. sativa*-japonica [54]. The divergence between *O. sativa*-japonica lineage and *S. bicolor*, *B. distachyon*, *L. perrieri*, or *O. sativa*-indica were respectively dated to *K*_s_ = 1.4, 1.2, 0.7, and 0.4. The rate of gene conversion events among ρ WGD-derived gene pairs decelerated over time compared with shortly after WGD. The number of gene conversion events is 58 after the divergence of *O. sativa*-japonica and *S. bicolor*, about fivefold higher than the number after the (much more recent) divergence of *O. sativa*-japonica and *O. sativa*-indica (Fig. 7c, d).

The proportion of tandem or proximal gene pairs experiencing gene conversion is more than that for other modes of gene duplication for model plants *Arabidopsis* and rice (Fig. 7e, f). In *Arabidopsis*, the percentage of converted TD-, PD-, TRD-, DSD-, and WGD-pairs is 20.6%, 0.0%, 0.5%, 2.4%, and 8.8% respectively. In rice, the percentage of converted TD-, PD-, TRD-, DSD-, and WGD-pairs is 23.0%, 17.5%, 0.6%, 2.2%, and 6.7% respectively. Rare gene conversion events were found in TRD-derived gene pairs, consistent with extensive sequence and expression divergence between TRD-duplicated genes.

### Inferring core gene families from 141 green plant genomes

The whole-genome protein sequences of 141 green plants containing 4,921,214 genes were used to construct core gene families by using OrthoFinder [55]. Large-scale BLASTP searches were carried out for each pair of 141 species. We identified 86,831 gene families (or orthologous groups) (freely available at figShare, 10.6084/m9.figshare.7264667.v1), including 4,333,638 (88.1%) genes, 6266 (18,889 genes, 0.4% of all genes) species-specific families, and 232 most conserved families (Additional file 7) in which all species have at least one gene. We found no strict single-copy gene families for these 141 species, which may be due to errors in genome annotation, frequent single-gene duplication, or pseudogenization. We further identified the most-preserved, intermediate-preserved, and least-preserved gene families in 141 plants. The most-preserved plant gene families are those orthologous groups in which all species must have at least one gene. The intermediate-preserved gene families are those orthologous groups in which the absence (or missing) of orthologous genes in up to three species was allowed. The least-preserved gene families are those orthologous groups in which the absence of orthologous genes in up to five species was allowed. Functional enrichment analysis for most-preserved, intermediate-preserved, and least-preserved plant gene families using *Arabidopsis* genes as a reference revealed that these genes were collectively enriched in GO terms involved in “membrane” and “organelle” such as plasma membrane, organelle part, nucleus, membrane−bounded organelle, intracellular organelle, and cytoplasmic part (Additional file 1: Figure S19 and Additional file 8). In addition, the enriched GO terms are also collectively involved in small GTPase-mediated signal transduction, nucleosome, cytoskeletal, light-harvesting complex, ATPase activity, actin filament-based movement.

**Fig. 8:**
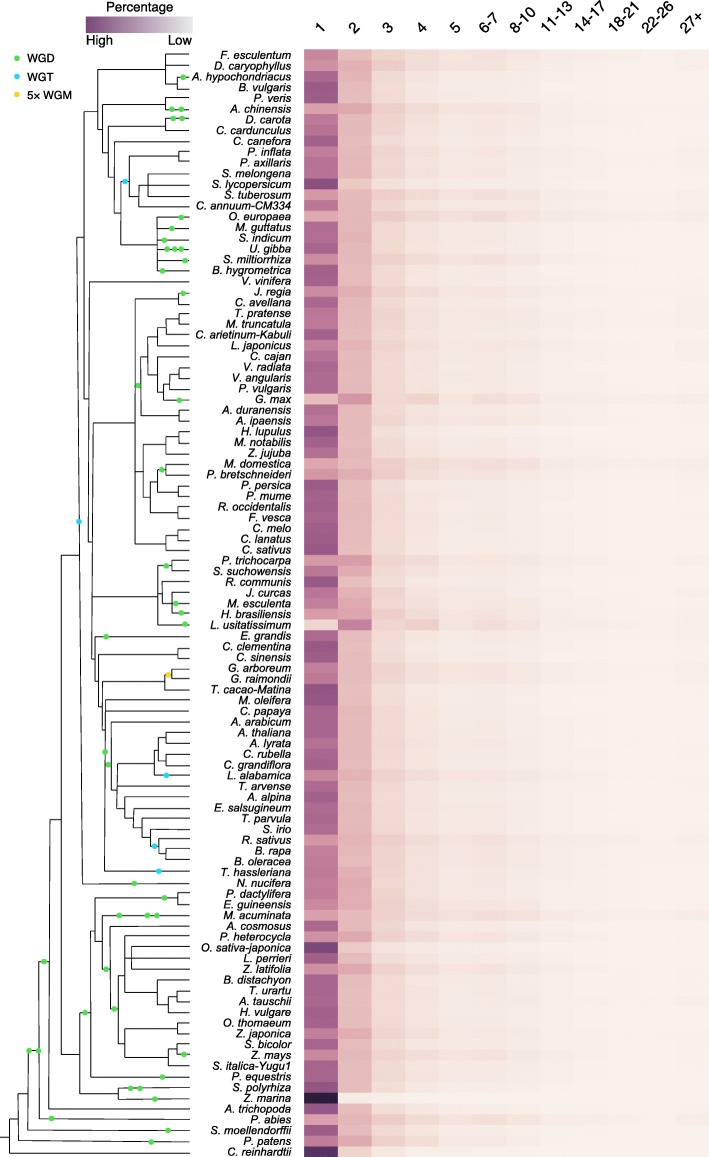
The distribution of gene family sizes across 141 plants. For each species, we calculated the percentage of gene families of a given size with respect to the total number of all gene families in this species. The top labels of the *x* axis indicate the gradient of different sizes of gene family

We further assigned the genes in each orthogroup into each single species and acquired the repertoire of gene families for each species (freely available on FigShare, DOI: 10.6084/m9.figshare.7264667). For each species, we calculated the percentage of gene families of a given size with respect to the total number of all gene families in this species, then investigated the distribution of gene family size in all plants (Fig. 8). A large percentage of small gene families (one to three members) were observed across all plants, showing a strong bias toward single-copy status. In green algae, the majority of gene families contained only one gene. For example, in *C. reinhardtii*, the proportion of single-gene families is 81.4%. The highest proportion (95.8%) of single-copy gene families was found in the marine angiosperm *Zostera marina*, forming a sharp contrast with closely related *Z. muelleri* with only 20.6% single-copy gene families. Species influenced by recent WGD or WGT, such as soybean (*Glycine max*), apple (*Malus domestica*), flax (*L. usitatissimum*), banana (*Musa acuminata*), and maize (*Z. mays*), possess more gene families of moderate number than other plants.

## Discussion

Classification and comparison of the five major types of gene duplication in 141 plant genomes affected by a diverse set of whole-genome multiplications spanning more than 100 million years provides new insight into genome evolution and biological innovation. Whole-genome duplication increases all genes in a genome in a balanced manner that may favor modification of entire pathways and processes [56] and is associated with longer half-lives of the resulting gene duplicates [27]. However, it is unclear whether these advantages outweigh the relatively constant availability of new tandem and proximal duplicates that may be important for plants to adapt to dramatic environmental changes [45, 57–60]. The C4 photosynthetic pathway, thought to have been an adaptation to hot, dry environments or CO_2_ deficiency [61–64] and independently appearing at least 50 times during angiosperm evolution [65, 66], includes some elements resulting from WGD and others from single-gene duplication, despite that all were in principle available from WGD in a cereal common ancestor [33]. Indeed, we found that the *K*_s_ peaks for WGD, transposed, and dispersed duplicates commonly overlapped in the same plant, suggesting that whole-genome duplication was also accompanied by extensive transposed and dispersed gene duplication, consistent with a recent study showing extensive relocation of *γ* duplicates shortly after the *γ* WGT event in core eudicots [48].

Different classes of gene duplicates showed distinct patterns of temporal and functional evolution. WGD-derived duplicates are more conserved with smaller *K*_a_/*K*_s_ ratios than tandem and proximal duplicates, suggesting that they have experienced long-term purifying selection. Proximal and tandem duplicates preserved in modern genomes, with relatively high *K*_a_/*K*_s_ ratios but relatively small *K*_s_ values per se, appear to experience more rapid functional divergence than other gene classes—supporting that positive selection plays an important role in the early stage of duplicate gene retention [67–69]. While concerted evolution may preserve homogeneity of tandem or proximal duplicates to a greater degree than genes that are distant from one another, this is not incompatible with rapid functional divergence [38].

Paralleling sequence divergence, expression divergence of duplicated genes gradually increases with age. Transposed duplicates preserved in modern genomes have high percentage of expression divergence in nearly all investigated species; this is consistent with both their antiquity and the nature of their evolution, with novel copies potentially being separated from *cis*-regulatory sequences at the original site and/or exposed to different ones at the new site. Environmental factors may accelerate expression divergence between duplicate genes [70], and frequent occurrence of transposed duplication may be important for plants to adapt to dramatic environmental changes [45, 57–60]. Physically linked (or tandem) duplications show generally less expression divergence than distant duplications, a result supported by many prior studies, e.g., [43, 44, 71–74]. Indeed, physically linked genes in the same paralogon (or syntenic block) are preferentially retained in *cis*-PPIs (protein–protein interactions) after WGD [75, 76].

Two types of subfunctionalization (SF) have been proposed [77–80]. One type of subfunctionalization takes place by complementary coding sequence changes between duplicated genes, leading to their functional divergence at the protein level, and eventually resulting in division of multiple functions of the progenitor gene. However, divergence at the biochemical level between two copies is limited even over long evolutionary times. The other type of subfunctionalization occurs by complementary loss or degenerative mutation of *cis*-regulatory elements between duplicated genes, creating inter-dependence between partially degenerated copies to maintain the full expression profiles of the ancestral gene in different tissues and/or conditions (defined as expression subfunctionalization (ESF)) [43, 78]. Many previous studies revealed that expression divergence between duplicate genes often occurred quickly after gene duplication [47, 81–84]. In this study, widespread divergence between expression profiles of duplicated genes was found in different modes of gene duplication—this can be largely explained by the expression subfunctionalization (or subfunctionalization) models, under which two duplicate genes evolved toward the partitioning of ancestral gene expression profiles in different tissues or conditions. The expression neofunctionalization (ENF) hypothesis, that one of the two gene copies gains a new *cis*-regulatory element in its promoter region and expresses in a new tissue, could also result in divergent expression profiles between duplicated genes such as some observed in this study [43, 85].

Among the earliest changes following polyploidization is gene conversion, nonreciprocal recombination between alleles or paralogous loci which homogenizes paralogous sequences or even chromosomal regions [86–89]. Gene conversion appears to occur virtually immediately in synthetic polyploid *Arachis* (peanut) [90]—indeed, abundant gene conversion after hybridization or polyploidization plays an important role in maintaining genome stability in plants and fungi [5, 18, 91, 92]. We detected relatively abundant gene conversion events in TD-, PD-, and WGD-pairs, which may be associated with their reduced expression divergence. The TRD- and DSD-pairs may have escaped the constraints induced by gene conversion. The dynamic changes of gene conversion rate found in this study, being high shortly after polyploidization and declining over time, show that prior findings over about 1 MY of cotton evolution [53] are generally applicable to a wide range of taxa and polyploidization events. The extensive gene conversion events occurring immediately after gene or genome duplication homogenize paralogs for a period of time and maintain a higher probability of functional compensation between duplicated genes, buffering the phenotypic effect caused by loss of one of two members of a duplicated pair [93–95]. Evolutionary divergence between duplicate genes may be suppressed by extensive gene conversion events during the early stage of genome duplication; however, this is not incompatible with rapid functional divergence of the TD- or PD-derived gene pair [96].

## Conclusions

The sharp increase in the number of sequenced plant genomes has empowered investigation of key aspects of evolution by application of uniform techniques to taxa spanning hundreds of million years of divergence, including model and non-model, crop and non-crop, flowering and non-flowering, seed and non-seed, vascular and non-vascular, and unicellular and multicellular species. Building on many studies of individual genomes, the comprehensive landscape of different modes of gene duplication identified across the plant kingdom by virtue of the ability to compare 141 genomes provides a solid foundation for further investigating the dynamic evolution and divergence of duplicate genes and for validating evolutionary models underlying duplicate gene retention. The contributions of gene duplication to gene regulatory networks, epigenetic variation, morphological complexity, and adaptive evolution are intriguing subjects for further investigation by this approach.

## Methods

### Collecting genome datasets

In this study, the genome datasets of 141 plants were downloaded from multiple comprehensive databases such as Phytozome (v11), NCBI, Ensembl Plants, and many other individual genome databases. These 141 plant genomes sample diverse taxa ranging from unicellular green alga (Chlorophytes) to Bryophytes, Lycophytes, gymnosperms, and angiosperms. The detailed information of these 141 species and their data sources can be retrieved in Additional file 2. Only the transcript with the longest CDS was selected for further analysis when several transcripts were available for the same gene.

### Identifying gene duplications

The different modes of gene duplication were identified using the *DupGen_finder* pipeline (https://github.com/qiao-xin/DupGen_finder). Firstly, the all-versus-all local BLASTP was performed using protein sequences (*E* < 1e^−10^, top 5 matches and m8 format output) to search all potential homologous gene pairs within each genome. Secondly, the *MCScanX* algorithm [97] was utilized to identify the WGD-derived gene pairs. Then, we excluded these WGD-pairs from the whole set of homologous pairs (or BLASTP hits) to further determine the single-gene duplications. If the two genes in a BLASTP hit that are adjacent to each other on the same chromosome, they were defined as tandem gene pair. Proximal gene pairs were defined as non-tandem pairs separated by 10 or fewer genes on the same chromosome. To identify transposed duplications, WGD, tandem, and proximal gene pairs were deducted from the whole set of homologous gene pairs. A transposed duplicate pair was required to meet the following criteria: one gene existed in its ancestral locus (named the parent copy) and the other was located in a non-ancestral locus (transposed copy). Two types of genes can be regarded as ancestral loci: (i) intra-species colinear genes and (ii) inter-species colinear genes. The intra-species colinear genes can be obtained from WGD-derived gene pairs, which have been identified above. Inter-species colinear genes were discerned by intergenomic synteny analysis, executing *MCScanX* on inter-species BLASTP files between the target and outgroup genomes. The sacred lotus (*Nelumbo nucifera*) and *Spirodela polyrhiza* were respectively taken as outgroup for all eudicot plants and all monocot plants to identify ancestral syntenic blocks. *Amborella trichopoda* was adopted as outgroup for *N. nucifera* and *S. polyrhiza* to find ancestral syntenic blocks. Genes located in these conserved syntenic blocks were deemed to be ancestral loci. The rarity of syntenic blocks between green algae (Chlorophytes), Bryophytes, Lycophytes, and other plants hindered the identification of ancestral loci in these species by applying inter-species synteny analysis. Therefore, we constructed orthologous relationships among genes of these species with large evolutionary distances to deduce the conserved ancestral genes. To identify the ancestral loci in *P. patens* (a Bryophyte) and *S. moellendorffii* (a Lycophyte), OrthoFinder [55] and whole-genome protein sequences were used to infer orthogroups among these two species and five other species: *P. abies*, *S. polyrhiza*, *N. nucifera*, *Amborella trichopoda*, and *Arabidopsis thaliana*. Based on the above orthogroups, if a gene in *P. patens* or *S. moellendorffii* has an ortholog pair in at least two other lineages, it is considered ancient and likely to have been present in the common ancestor of land plants. Similarly, we built the orthogroups among eight green algae species to determine the ancestral loci within each green algae genome. Based on the above steps, BLASTP hits to both an ancestral and a novel locus were defined as transposed duplications. Finally, after removing WGD, tandem, proximal, and transposed duplications from the whole set of homologous gene pairs, the remaining gene pairs were classified as dispersed duplications. Noting that the same dispersed gene may have several BLASTP hits resulting in multiple gene pairs for one gene, we only considered the dispersed gene pairs with highest similarity in this situation.

For gymnosperm species, we applied an alternative method to infer gene duplications. In this study, we initially selected two reference gymnosperm species: *Picea abies* and *Pinus taeda*, both belonging to Pinaceae. However, no or few syntenic or colinear blocks could be detected within these two genomes due to the fragmented assembly; thus, we used an alternative strategy to find potential duplicate gene pairs derived from WGDs. A recent study suggested that Pinaceae lineages had experienced one ancient WGD shared with other seed plants corresponding to a *K*_s_ peak with a median *K*_s_ = 0.75 to 1.5 and one younger WGD in a Pinaceae ancestor corresponding to *K*_s_ peak with a median *K*_s_ = 0.2 to 0.4 [98]. According to the above results, we firstly selected duplicate gene pairs corresponding to these two putative WGD peaks in the *K*_s_ age distribution from all-blast-all output. Furthermore, we identified orthogroups among genes from *P. abies*, *P. taeda*, and three other Pinaceae species (*Pinus lambertiana*, *Pseudotsuga menziesii*, and *Picea glauca*) by using the OrthoFinder software [55], which utilize a novel method to infer orthogroups of protein coding genes and is suitable for orthogroup inference from incomplete genome assemblies. Based on the above two steps, if each gene of a duplicate pair from the aforementioned two *K*_s_ peaks in *P. abies* or *P. taeda* has an ortholog pair in at least two other lineages, we assumed that this duplicate pair was created by WGDs in a common Pinaceae ancestor rather than independently in each lineage. By using the same rules applied in other plants, the tandem and proximal gene pairs were identified in *P. abies* or *P. taeda*. Based on orthogroups among five gymnosperm species, if a gene in *P. abies* or *P. taeda* has an ortholog pair in at least two other lineages, it is considered ancient and likely to have been present in the common ancestor of Pinaceae species. Then, we determined the transposed gene pairs comprised of an ancestral and a novel locus after excluding the WGD, tandem, and proximal gene pairs from the population of BLASTP hits. At last, the remaining gene pairs after removing other modes of gene duplications from BLASTP hits were classified as dispersed gene pairs.

### Calculating *K*_a_, *K*_s_, and *K*_a_/*K*_s_ values

For each duplicate gene pair, we aligned their protein sequences using MAFFT (v7.402) [99] with the L-INS-i option and converted the protein alignment into a codon alignment using PAL2NAL [100]. Then, the resulting codon alignment was formatted into an AXT format using a custom Perl script. *γ*-MYN method (a modified version of the Yang–Nielsen method) [101, 102] incorporated in KaKs_Calculator 2.0 [103] was used to calculate *K*_a_ and *K*_s_ values by implementing the Tamura–Nei model [104]. The *K*_s_ values > 5.0 were excluded from further analysis due to the saturated substitutions at synonymous sites [105, 106]. The pipeline used to calculate *K*_a_ and *K*_s_ values is freely available on GitHub (https://github.com/qiao-xin/Scripts_for_GB).

### RNA-seq data and quantification

Single-end or paired-end RNA-seq reads were downloaded from NCBI SRA (https://www.ncbi.nlm.nih.gov/sra). The RNA-seq samples used in this study were documented in Additional file 6. The raw reads were filtered using Trimmomatic (version 0.36) (http://www.usadellab.org/cms/?page=trimmomatic). We filtered the raw reads according to the following procedure: (1) removing adapters (pair-end: ILLUMINACLIP:TruSeq3-PE.fa:2:30:10 and single-end: ILLUMINACLIP:TruSeq3-SE:2:30:10); (2) removing leading low quality or N bases (below quality 15) (LEADING:15); (3) removing trailing low quality or N bases (below quality 15) (TRAILING:15); (4) scanning the read with a 4-base wide sliding window, cutting when the average quality per base drops below 15 (SLIDINGWINDOW:4:15); and (5) dropping reads below 55 or 36 bases long (pair-end: MINLEN:55 and single-end: MINLEN:36). Next, the abundances of transcripts from RNA-Seq data were estimated using kallisto [107]. The reference transcripts obtained from genome annotation files were used to build kallisto indices. Then, the kallisto quantification algorithm was performed with default parameters (for single-ends, -l 200 -s 20) to process either single-end or paired-end reads, outputting the normalized count estimates and TPM (transcripts per million) values for each transcript. The TPM value was used as the measure of expression level of the genes in different tissues and conditions.

We further extracted all intergenic regions at the whole-genome level for investigated species and quantified their expression abundances using the same procedure and RNA-seq reads used for exonic regions. The medians of the distributions of TPM values for intergenic sequences in different tissues and conditions are close to 0. Therefore, we used the mean value of the medians (the 50th percentile) obtained from the TPM distributions for intergenic sequences in different tissues and conditions as the threshold of expression (Additional file 1: Figure S20 and S21).

### Estimating expression divergence

Duplicated gene pairs in which both gene copies were expressed in at least one tissue or development stage were used to calculate Pearson correlation coefficients (*r*) between expression profiles of the two gene copies. The two genes in a random pair should have unrelated function and differential expression, so we can determine the cutoff for divergent expression by comparing distributions of *r* values for random gene pairs to those for duplicated gene pairs. We randomly selected 10,000 gene pairs from each species and computed *r* values between their expression profiles. We determined a cutoff from the distribution of *r* values for random gene pairs in each species and required that 95% of the *r* values obtained from the distribution be less than this cutoff value. The duplicated gene pairs with *r* less than this cutoff can be considered to have diverged in expression (Additional file 1: Figure S15 and S16).

### Detecting gene conversion

The method used to detect gene conversion is as described in former studies [89, 108, 109]. Firstly, we identified homologous gene quartets, comprised of two paralogs in the species of interest and their respective orthologs in outgroup species. For *Arabidopsis*, *Aethionema arabicum*, *Eutrema salsugineum*, *Capsella rubella*, and *Arabidopsis lyrata* were used as outgroup species. For rice (*O. sativa* L. (ssp. japonica)), *Sorghum bicolor*, *Brachypodium distachyon*, *Leersia perrieri*, and *Oryza sativa* L. (ssp. indica) were used as outgroup species. The number of homologous gene quartets identified between *Arabidopsis* and the four outgroup species *A. arabicum*, *E. salsugineum*, *C. rubella*, and *A. lyrata* are 615, 1165, 1355, and 788 respectively. The number of homologous gene quartets identified between rice and the four outgroup species *S. bicolor*, *B. distachyon*, *L. perrieri*, and *O. sativa*-indica are 761, 718, 917, and 1140. To identify the gene conversion events in different modes of duplicated gene pairs, we chose *A. arabicum* and *S. bicolor* as outgroups for *Arabidopsis* and rice respectively to determine homologous gene quartets. The frequency of gene conversion events for different modes of duplicated gene pairs was determined in model plants *Arabidopsis* and rice. Then, we compared gene similarity or tree topology between homologs in quartets by estimating synonymous nucleotide substitution rates (*K*_s_) between them. We performed a bootstrap test to evaluate the significance of putative gene conversions with 1000 repetitive samplings to produce a bootstrap frequency indicating the confidence level of the supposed conversion [89, 108]. The pipelines used to identify homologous gene quartets and detect gene conversion are available on GitHub (https://github.com/qiao-xin/Scripts_for_GB/tree/master/detect_gene_conversion). All homologous gene quartets identified in this study have been deposited on FigShare (10.6084/m9.figshare.7264667.v1).

### Inferring the orthogroups of 141 green plants

The OrthoFinder [55] algorithm was utilized to construct the orthogroups for the 141 plants. It has been demonstrated that the OrthoFinder is more accurate and faster than other commonly used orthogroup inference methods such as OrthoMCL [55, 110]. To run OrthoFinder with pre-computed BLAST results, we performed all-vs-all BLASTP searches (*E* < 1e^−10^, top 5 matches and m8 format output) for each pairwise genome comparison between species and self-genome comparisons by using protein sequences. Then, we ran OrthoFinder with default parameters using the BLASTP outputs as inputs and obtained a file containing the orthologous groups (or gene families) of genes from these 141 species. Furthermore, we assigned the genes in each orthogroup into each single species and acquired the repertoire of gene families for each species (freely available on FigShare, 10.6084/m9.figshare.7264667.v1). We then investigated the distribution of gene family size in all studied plants.

### Gene ontology enrichment analysis

Because the members of a gene family have similar functions, we only conducted the functional enrichment analysis for the *Arabidopsis* gene sets from the most-conserved, intermediate-conserved, and least-conserved gene families (or orthogroups) in 141 plants. Firstly, we retrieved all *Arabidopsis* genes from most-preserved, intermediate-preserved, and least-preserved gene families respectively. GO annotations for the genes in *Arabidopsis* were downloaded from Phytozome11 (https://phytozome.jgi.doe.gov/pz/portal.html). Furthermore, we detected the overrepresented GO slim terms in these *Arabidopsis* genes by using the GOATOOLS package [111]. The *P* values used to evaluate significant enrichment of certain GO terms were calculated based on Fisher’s exact test and corrected by an FDR test correction method (false discovery rate implementation using resampling). Finally, we used corrected *P* value < 0.01 as the threshold to determine significant overrepresentation of certain GO terms.

## Additional files

Additional file 1: Supplementary Table S1 and Figure S1-21. (DOCX 5373 kb)

Additional file 2: The detailed information of 141 plant species used in this study. (XLSX 76 kb)

Additional file 3: The absolute number of different modes of duplicate gene pairs in each taxon. (XLSX 49 kb)

Additional file 4: The fitted *K*_s_ peak for WGD genes in each species. (XLSX 62 kb)

Additional file 5: The enriched GO terms for tandem and proximal duplicate genes in *Arabidopsis thaliana*. (XLSX 69 kb)

Additional file 6: The list of all RNA-Seq samples collected from different plants investigated in this study. (XLSX 105 kb)

Additional file 7: The 232 most conserved gene families in 141 plants. (XLSX 1968 kb)

Additional file 8: The enriched GO terms for the most-preserved, intermediate-preserved and least-preserved gene families in 141 plants. (XLSX 1018 kb)
