# Supplementary material for: Gene duplication and evolution in recurring polyploidization–diploidization cycles in plants
Source: Genome Biol. 2019 Feb 21;20:38. doi: 10.1186/s13059-019-1650-2 (PMC6383267; doi:10.1186/s13059-019-1650-2)
Supplement: Supplementary file 1 — Supplementary Table S1 and Figure S1-21. (DOCX 5373 kb) [file 13059_2019_1650_MOESM1_ESM.docx]

**Supplementary Tables**

**Table S1. The percentage of duplicated gene pairs under different selective pressure in seven model species.**

| Species | Selective pressure | WGD (%) | Tandem (%) | Proximal (%) | Transposed (%) | Dispersed (%) |
| --- | --- | --- | --- | --- | --- | --- |
| *Arabidopsis thaliana* | purifying | 100 | 96.5 | 94.9 | 99.7 | 99.3 |
|  | positive | 0.0 | 3.3 | 5.1 | 0.3 | 0.6 |
| *Oryza sativa* | purifying | 99.4 | 98.7 | 98.3 | 99.7 | 98.5 |
|  | positive | 0.5 | 1.2 | 1.5 | 0.2 | 1.4 |
| *Amborella trichopoda* | purifying | 96.3 | 91.9 | 92.5 | 97.2 | 94.5 |
|  | positive | 3.3 | 7.7 | 7.2 | 2.7 | 5.2 |
| *Picea abies* | purifying | 98.8 | 86.2 | 90.9 | 89.7 | 94.2 |
|  | positive | 1.1 | 13.2 | 9.1 | 9.9 | 5.5 |
| *Selaginella moellendorffii* | purifying | 91.8 | 94.3 | 92.2 | 98.3 | 96.4 |
|  | positive | 7.6 | 5.2 | 7.5 | 1.6 | 3.4 |
| *Physcomitrella patens* | purifying | 99.7 | 91.4 | 93.0 | 99.7 | 99.2 |
|  | positive | 0.3 | 8.4 | 7.0 | 0.3 | 0.8 |
| *Chlamydomonas reinhardtii* | purifying | 98.2 | 98.1 | 98.1 | 95.7 | 98.7 |
|  | positive | 1.8 | 1.7 | 1.9 | 4.3 | 1.2 |

**Supplementary Figures**


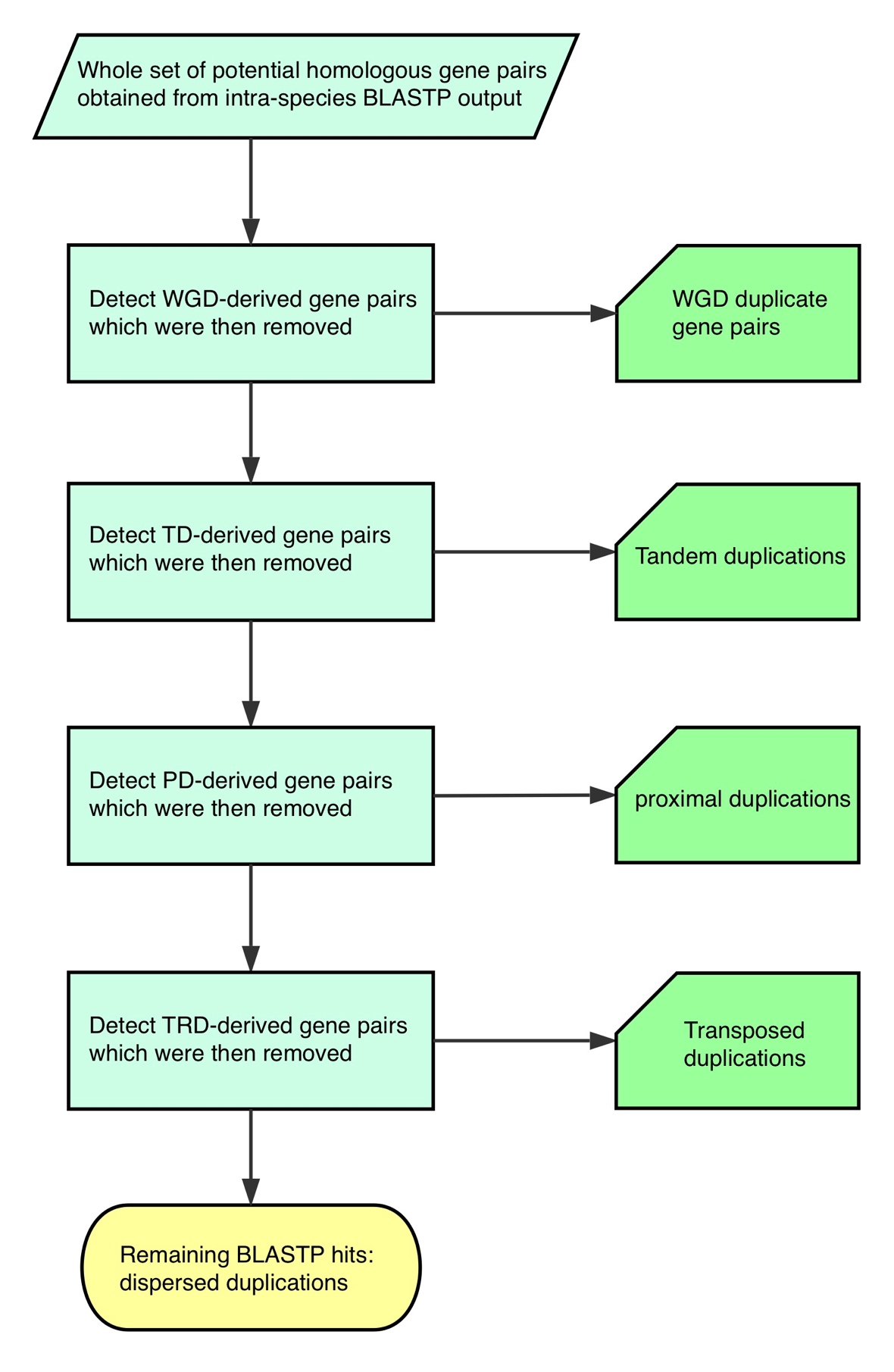


**Figure S1. The schematic diagram of *DupGen_finder* pipeline.**


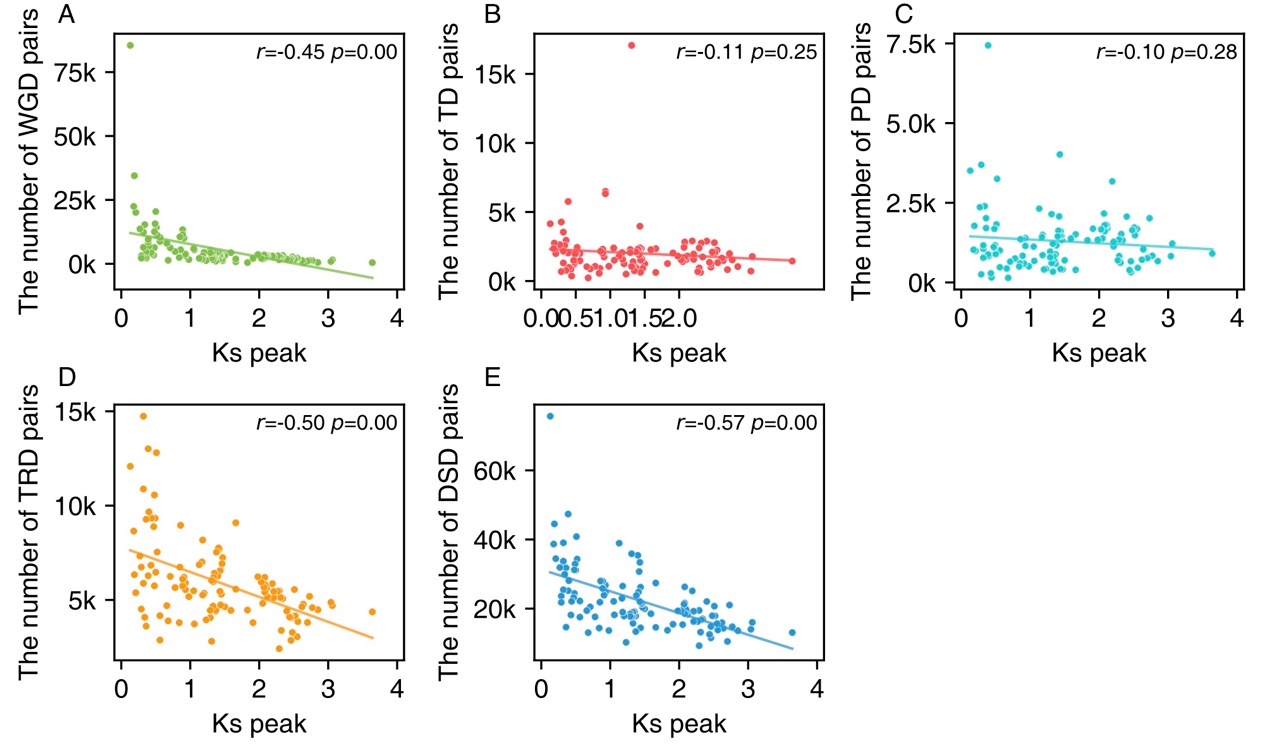


**Figure S2. The linear regression between the number of different types of gene pairs and Ks peak of WGD genes from different taxa.**

(A) WGD-pairs. (B) TD-pairs. (C) PD-pairs. (D) TRD-pairs. (E) DSD-pairs. The linear regression analysis was performed and Pearson correlation coefficient (*r*) was annotated in each subplot. Those taxa with abnormal Ks peaks were excluded due to fragmented assembly.


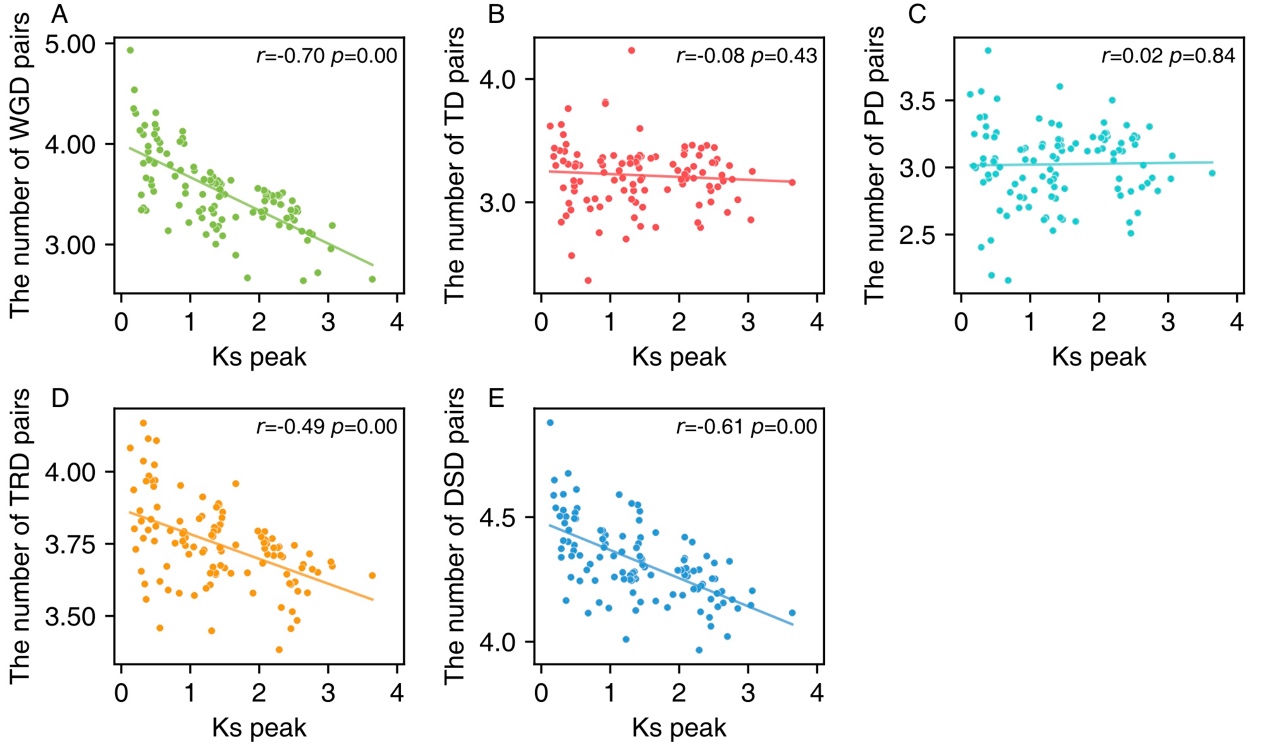


**Figure S3. The linear regression between the log10-transformed number of different types of gene pairs and Ks peak of WGD genes from different taxa.**

(A) WGD-pairs. (B) TD-pairs. (C) PD-pairs. (D) TRD-pairs. (E) DSD-pairs. The linear regression analysis was performed and Pearson correlation coefficient (*r*) was annotated in each subplot. Those taxa with abnormal Ks peaks were excluded due to fragmented assembly.


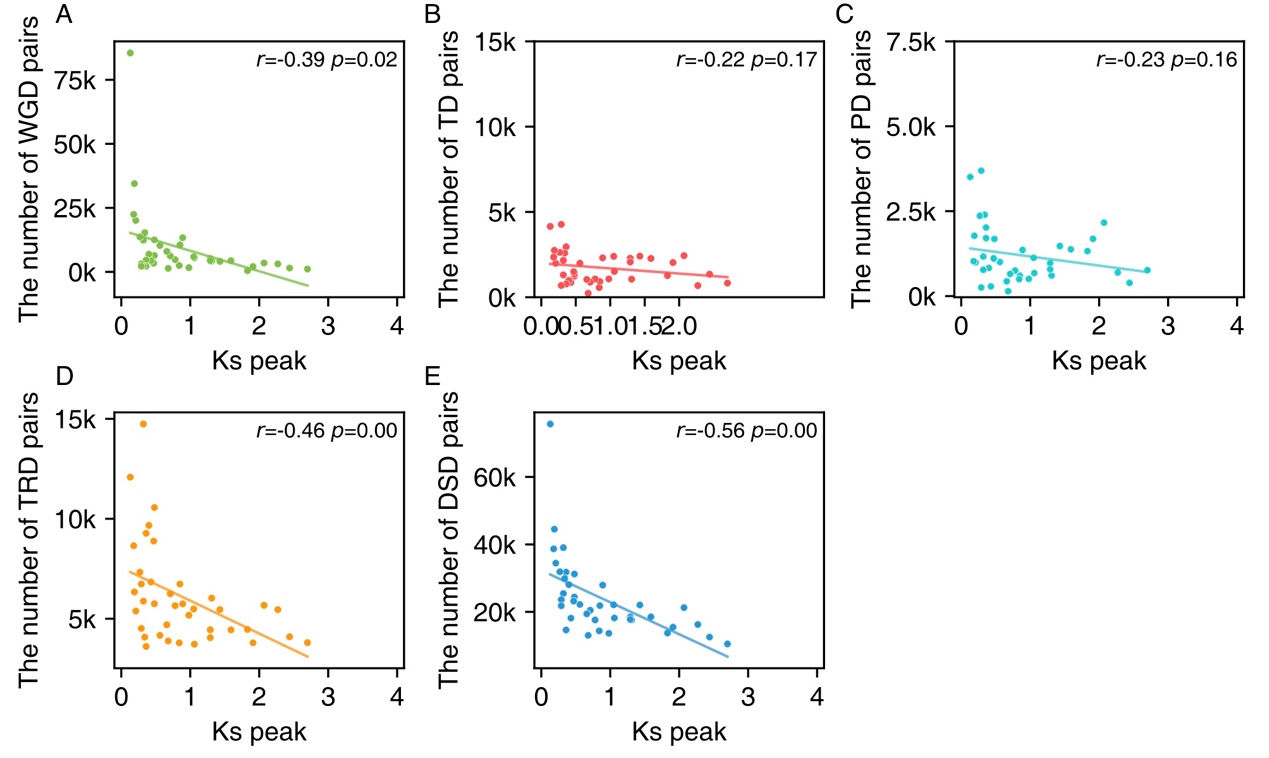


**Figure S4. The linear regression between the number of different types of gene pairs and Ks peak of WGD genes from different taxa.**

(A) WGD-pairs. (B) TD-pairs. (C) PD-pairs. (D) TRD-pairs. (E) DSD-pairs. The linear regression analysis was performed and Pearson correlation coefficient (*r*) was annotated in each subplot. Those taxa with abnormal Ks peaks were excluded due to fragmented assembly.


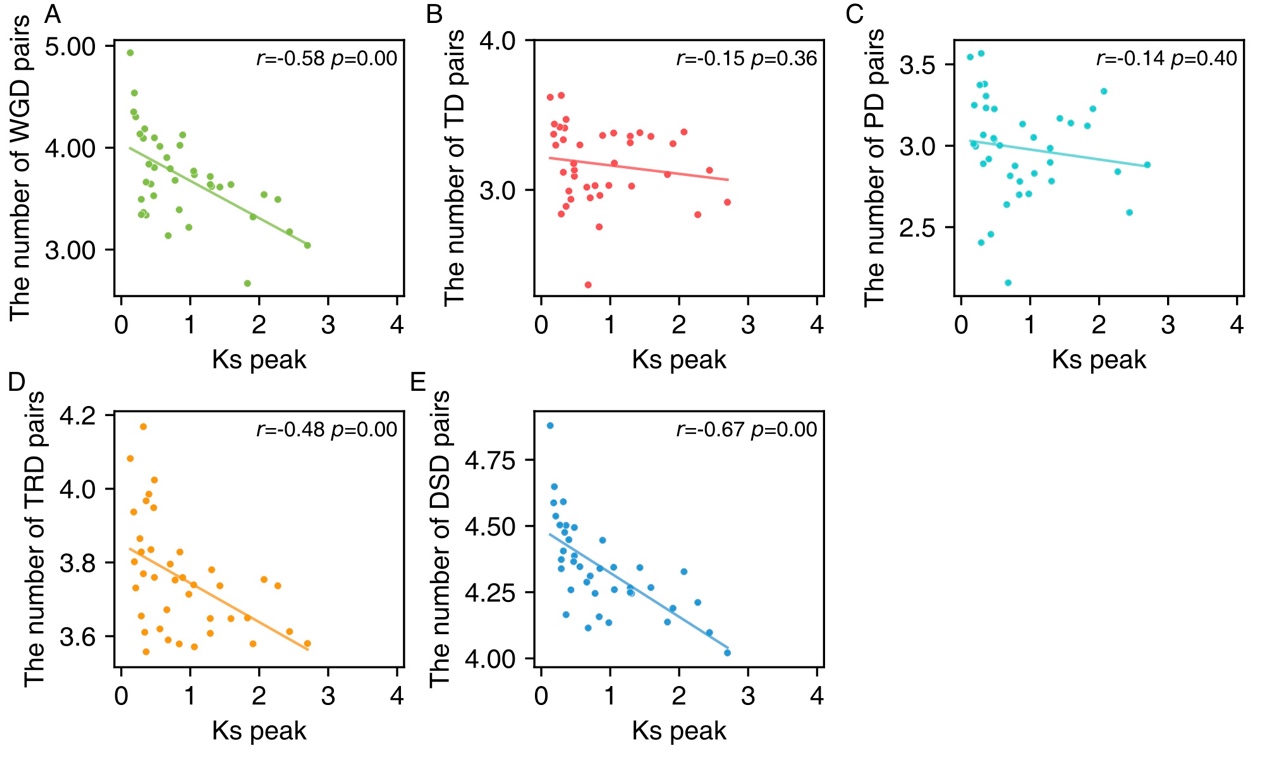


**Figure S5. The linear regression between the log10-transformed number of different types of gene pairs and Ks peak of WGD genes from different taxa.**

(A) WGD-pairs. (B) TD-pairs. (C) PD-pairs. (D) TRD-pairs. (E) DSD-pairs. The linear regression analysis was performed and Pearson correlation coefficient (*r*) was annotated in each subplot. Those taxa with abnormal Ks peaks were excluded due to fragmented assembly.


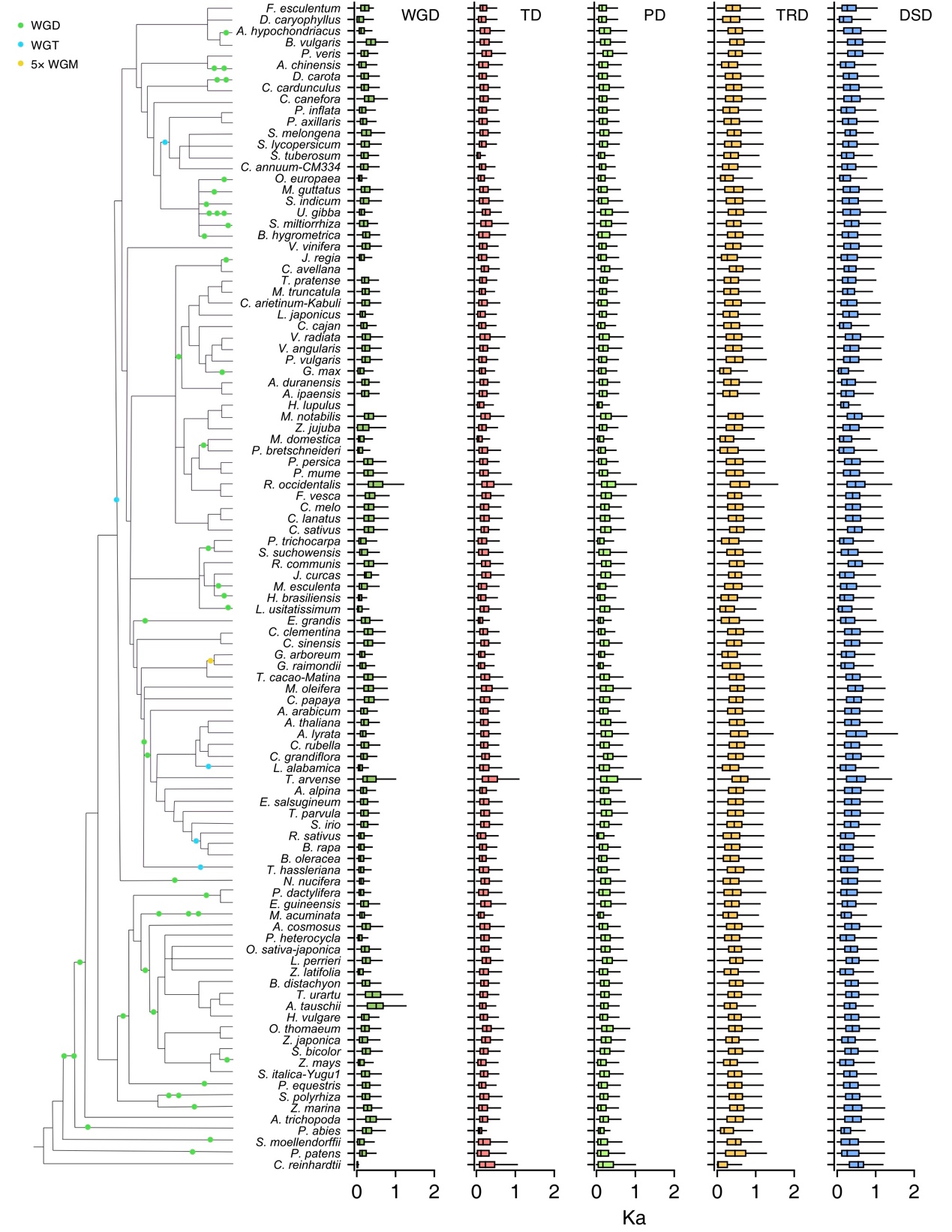


**Figure S6. The Ka distributions of gene pairs derived from different modes of duplication in representative plant genomes.**

WGD: whole-genome duplication; TD: tandem duplication; PD: proximal duplication; TRD: transposed duplication; DSD: dispersed duplication.


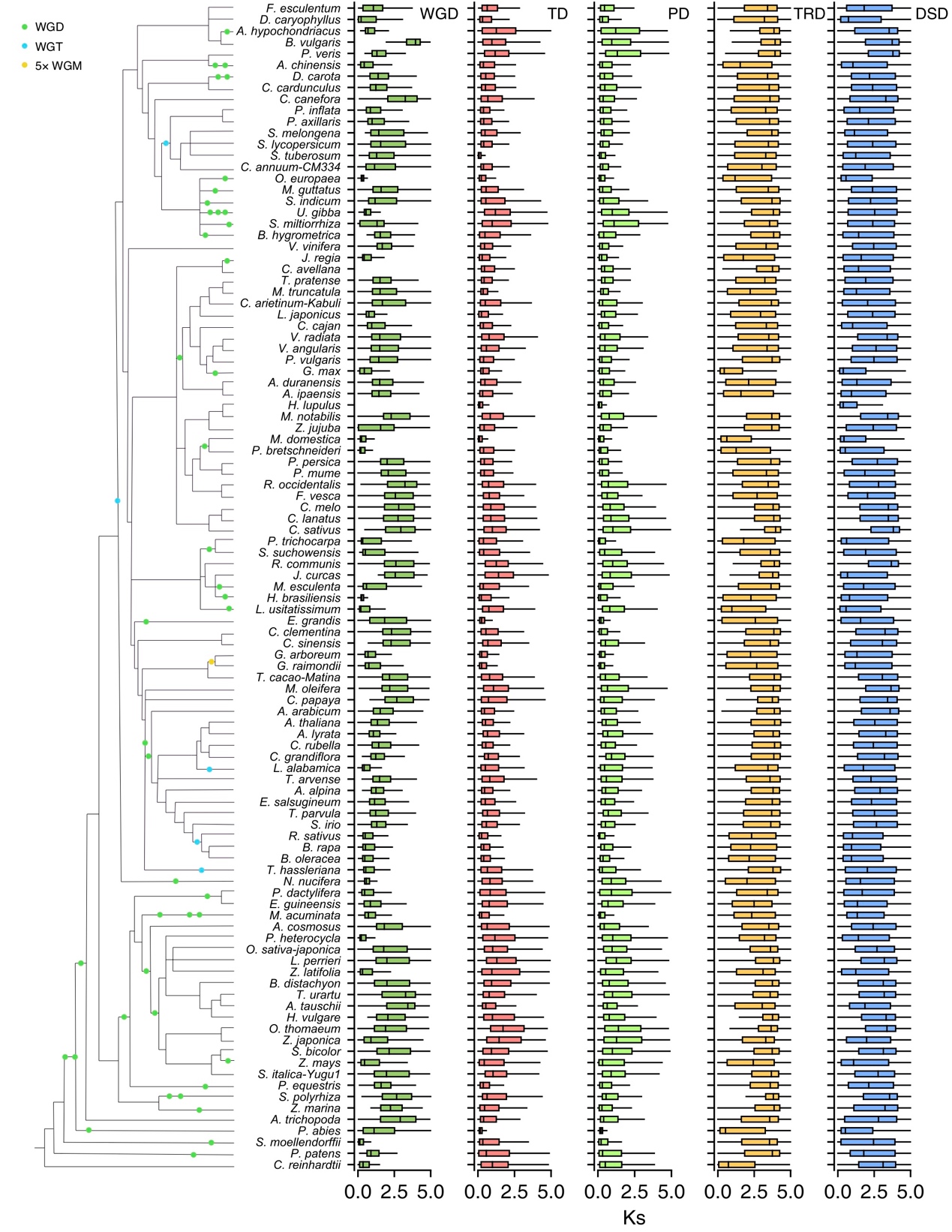


**Figure S7. The Ks distributions of gene pairs derived from different modes of duplication in representative plant genomes.**

WGD: whole-genome duplication; TD: tandem duplication; PD: proximal duplication; TRD: transposed duplication; DSD: dispersed duplication.


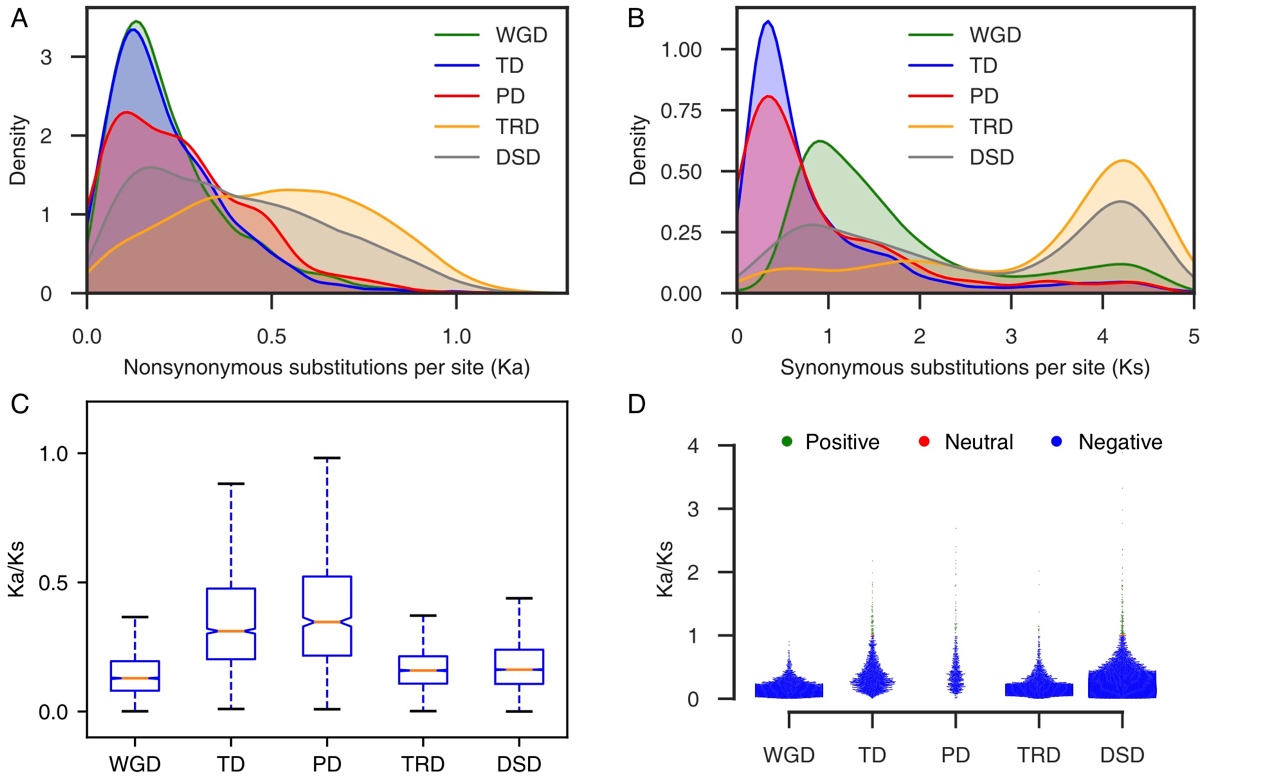


**Figure S8. Evolution of gene pairs duplicated by different modes in Arabidopsis.**

(A) Ka distributions; (B) Ks distributions; (C) Comparison of Ka/Ks ratio -- boxes represent the interquartile range (IQR), notches are used to show the 95% confidence interval (CI) for the median (orange line) and whiskers indicate variable ranges (1.5 * IQR); (D) Selection pressure.


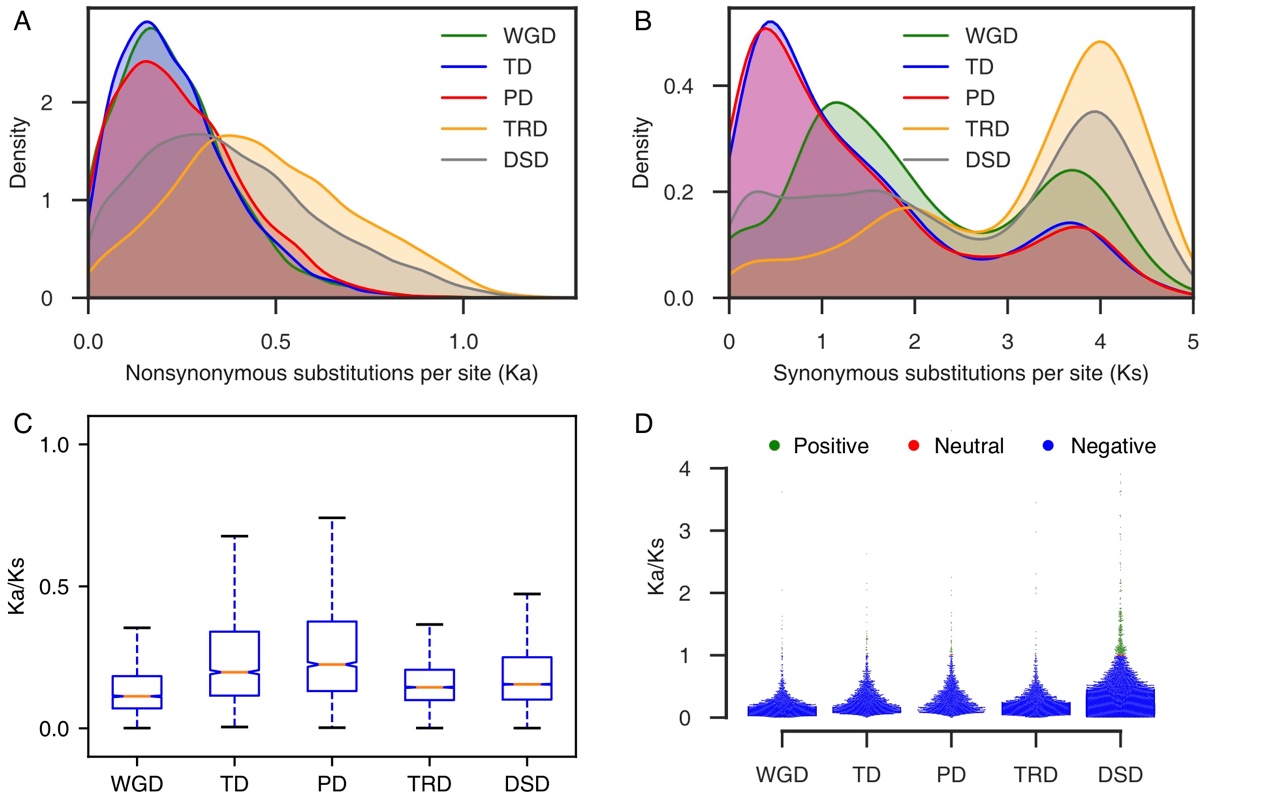


**Figure S9. Evolution of gene pairs duplicated by different modes in *Oryza sativa*.**

(A) Ka distributions; (B) Ks distributions; (C) Comparison of Ka/Ks ratio ratio -- boxes represent the interquartile range (IQR), notches are used to show the 95% confidence interval (CI) for the median (orange line) and whiskers indicate variable ranges (1.5 * IQR); (D) Selection pressure.


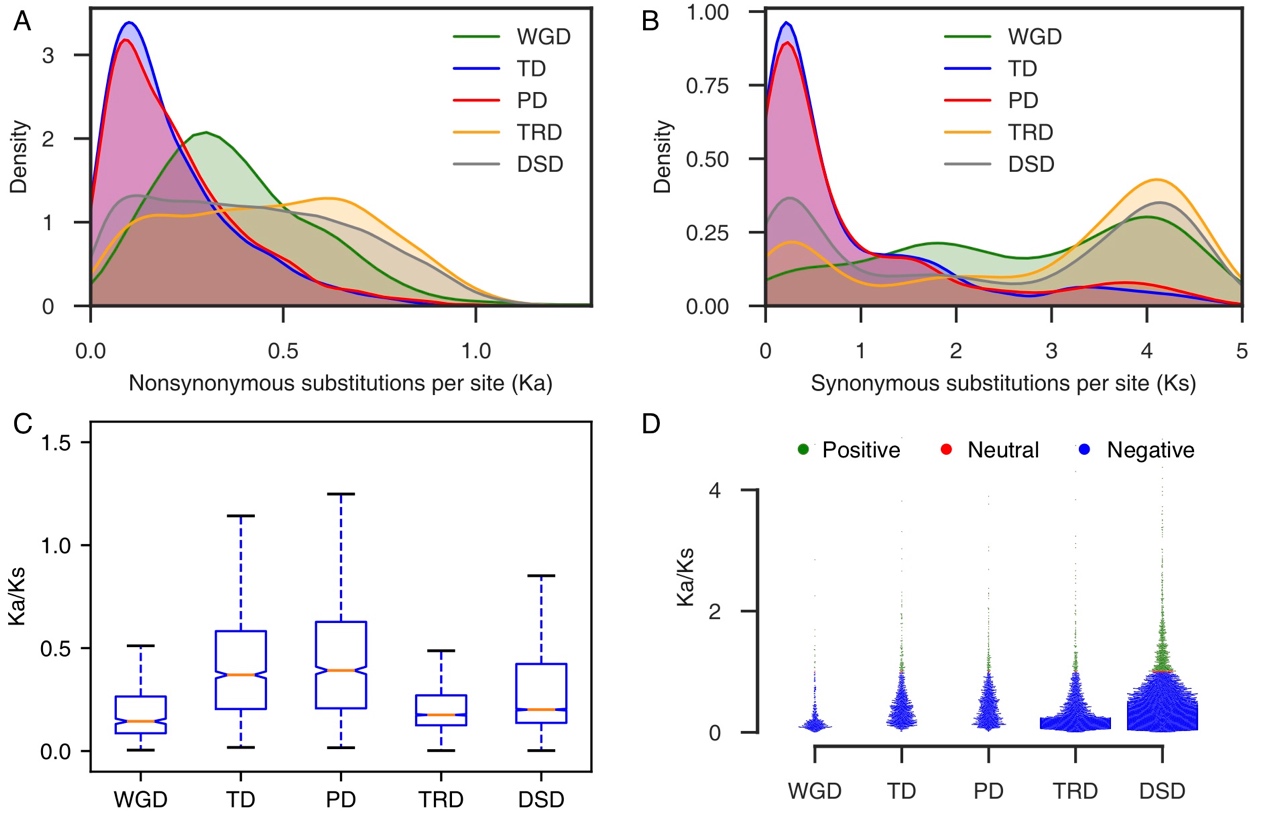


**Figure S10. Evolution of gene pairs duplicated by different modes in *Amborella trichopoda*.**

(A) Ka distributions; (B) Ks distributions; (C) Comparison of Ka/Ks ratio -- boxes represent the interquartile range (IQR), notches are used to show the 95% confidence interval (CI) for the median (orange line) and whiskers indicate variable ranges (1.5 * IQR); (D) Selection pressure.


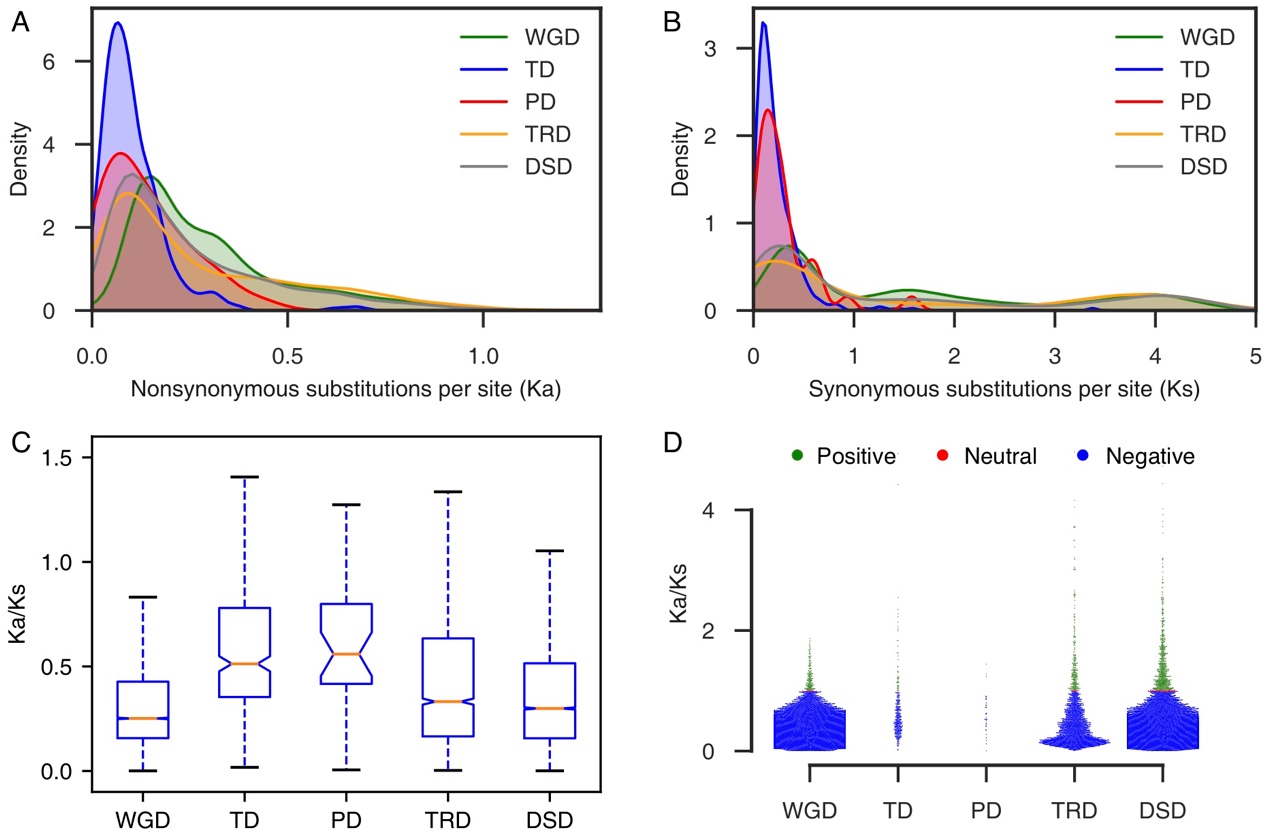


**Figure S11. Evolution of gene pairs duplicated by different modes in *Picea abies*.**

(A) Ka distributions; (B) Ks distributions; (C) Comparison of Ka/Ks ratio -- boxes represent the interquartile range (IQR), notches are used to show the 95% confidence interval (CI) for the median (orange line) and whiskers indicate variable ranges (1.5 * IQR); (D) Selection pressure.


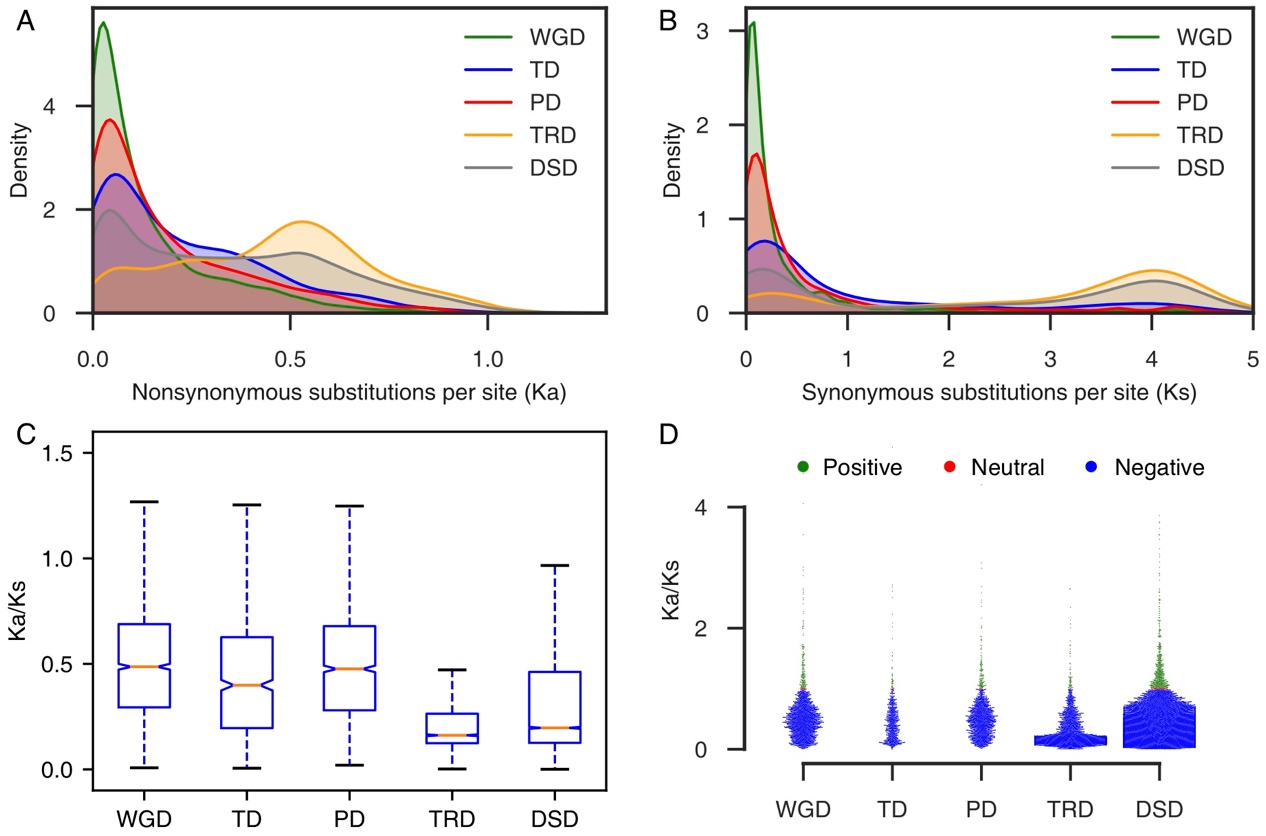


**Figure S12. Evolution of gene pairs duplicated by different modes in *Selaginella moellendorffii*.**

(A) Ka distributions; (B) Ks distributions; (C) Comparison of Ka/Ks ratio -- boxes represent the interquartile range (IQR), notches are used to show the 95% confidence interval (CI) for the median (orange line) and whiskers indicate variable ranges (1.5 * IQR); (D) Selection pressure.


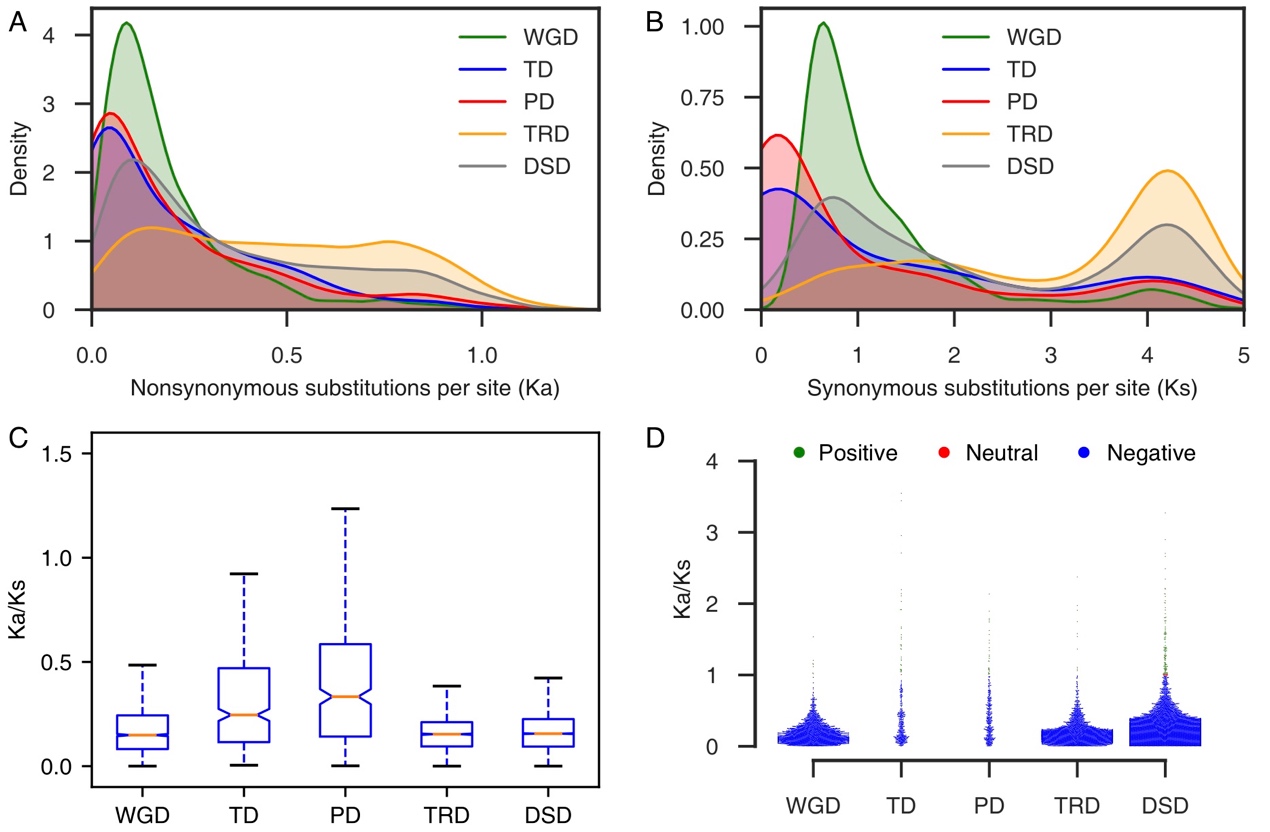


**Figure S13. Evolution of gene pairs duplicated by different modes in *Physcomitrella patens*.**

(A) Ka distributions; (B) Ks distributions; (C) Comparison of Ka/Ks ratio -- boxes represent the interquartile range (IQR), notches are used to show the 95% confidence interval (CI) for the median (orange line) and whiskers indicate variable ranges (1.5 * IQR); (D) Selection pressure.


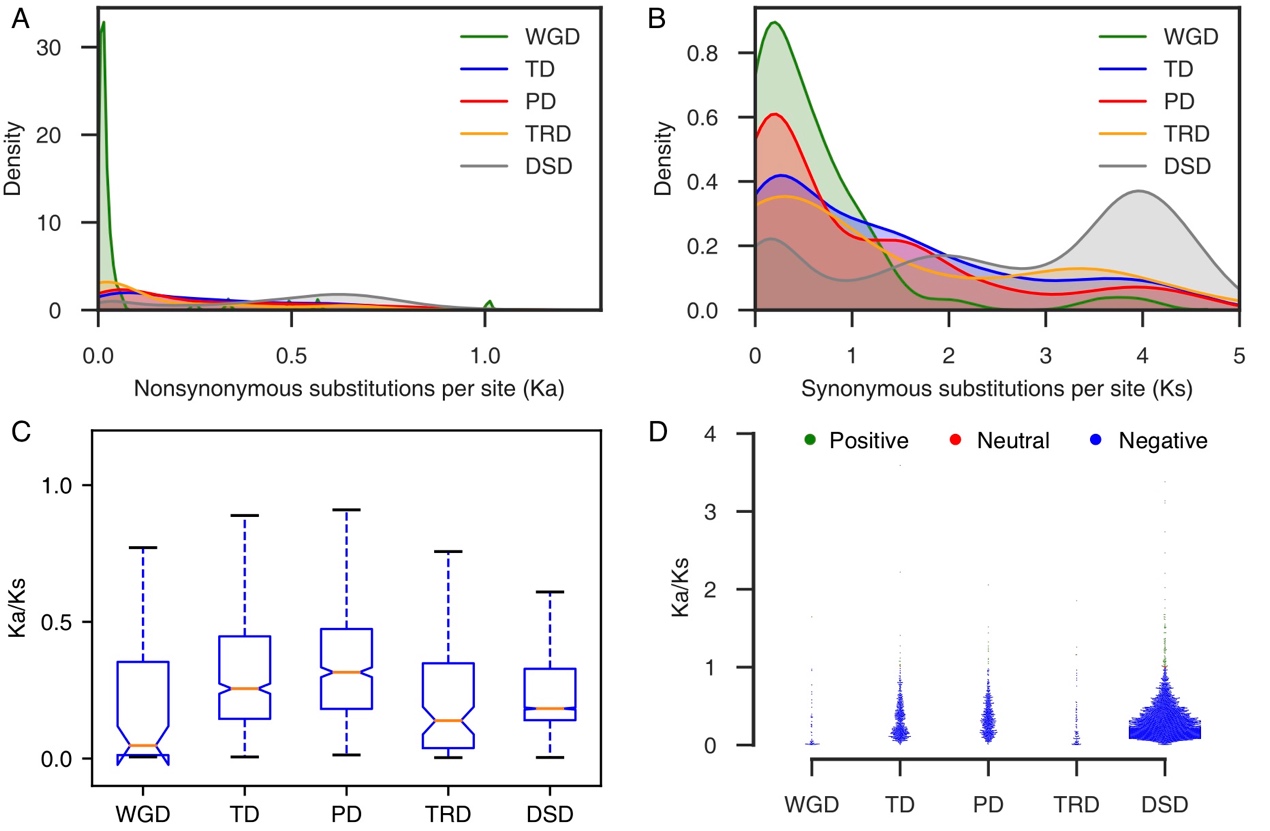


**Figure S14. Evolution of gene pairs duplicated by different modes in *Chlamydomonas reinhardtii*.**

(A) Ka distributions; (B) Ks distributions; (C) Comparison of Ka/Ks ratio -- boxes represent the interquartile range (IQR), notches are used to show the 95% confidence interval (CI) for the median (orange line) and whiskers indicate variable ranges (1.5 * IQR); (D) Selection pressure.


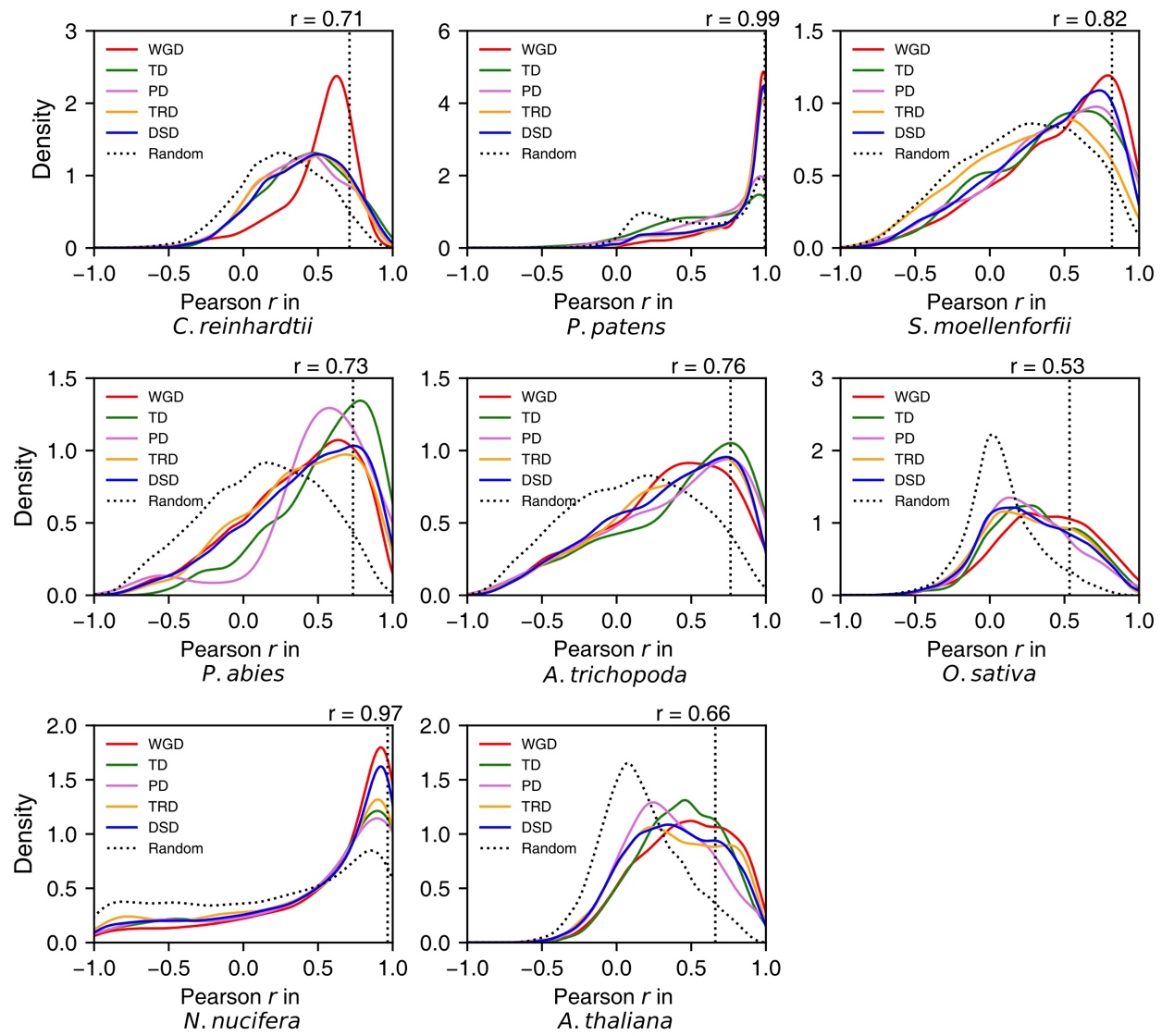


**Figure S15.** The density distributions of Pearson’s correlation coefficient (*r*) between the expression profiles of two copies derived from WGD, tandem duplication (TD), proximal duplication (PD), transposed duplication (TRD), and dispersed duplication (DSD) in (A) *C. reinhardtii*, (B) *P. patens*, (C) *S. moellendorffii*, (D) *P. abies*, (E) *A. trichopoda*, (F) *O. sativa*, (G) *N. nucifera*, and (H) *A. thaliana*. The vertical dotted line indicates the 95% quantile in the *r* values distribution for 10,000 random gene pairs.


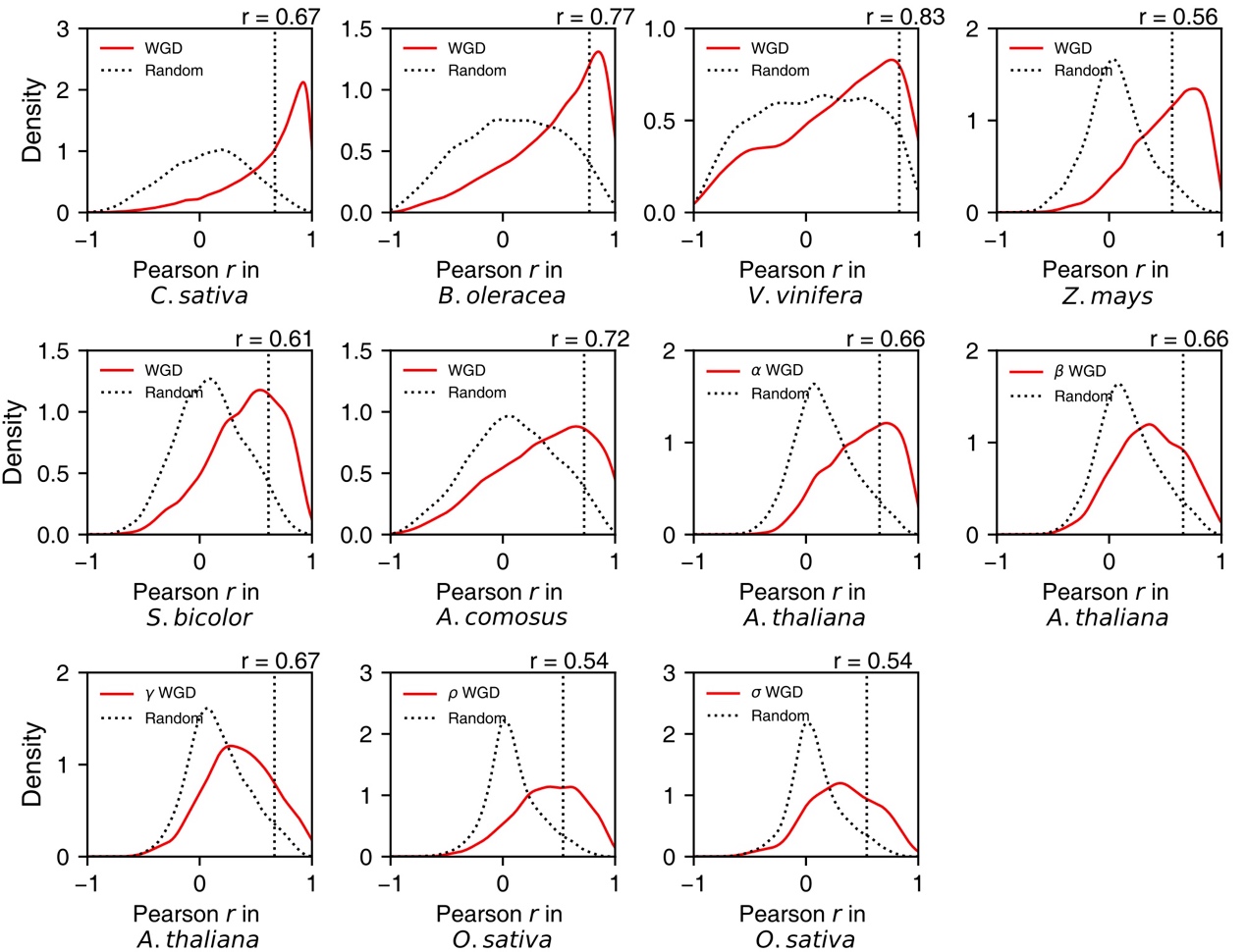


**Figure S16.** The density distributions of Pearson’s correlation coefficient (*r*) between the expression profiles of two copies derived from genome duplication events of different ages. (A) *C. sativa*, (B) *B. oleracea*, (C) *V. vinifera*, (D) *Z. mays*, (E) *S. bicolor*, (F) *A. comosus*, (G-I) Arabidopsis alpha (α)-beta (β)-gamma (γ), (J, K) Rice rho (ρ)-sigma (σ).


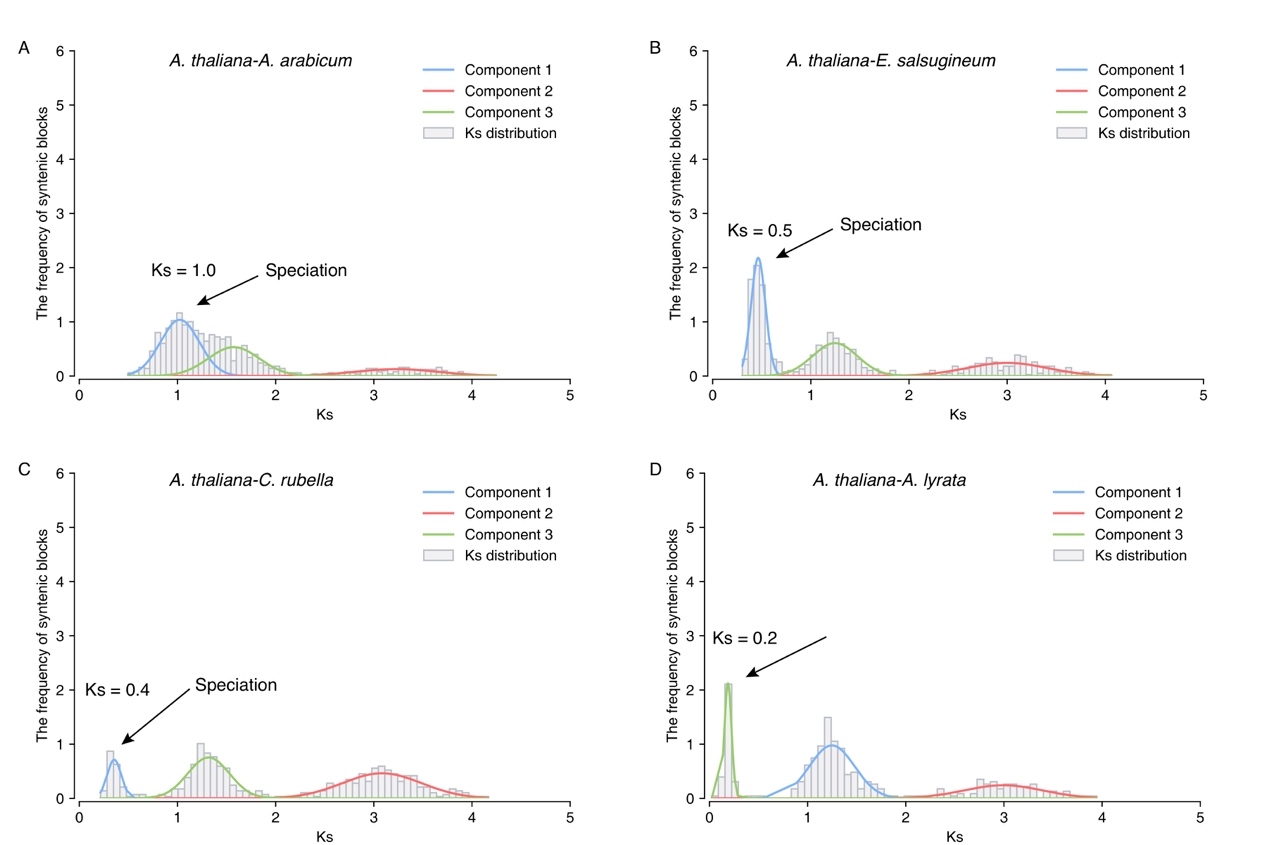


**Figure S17.** The estimation of time of speciation between *A. thaliana* and *Aethionema arabicum*, *Eutrema salsugineum*, *Capsella rubella*, or *Arabidopsis lyrata* respectively. The Ks distribution was fitted using Gaussian mixture models (GMM).


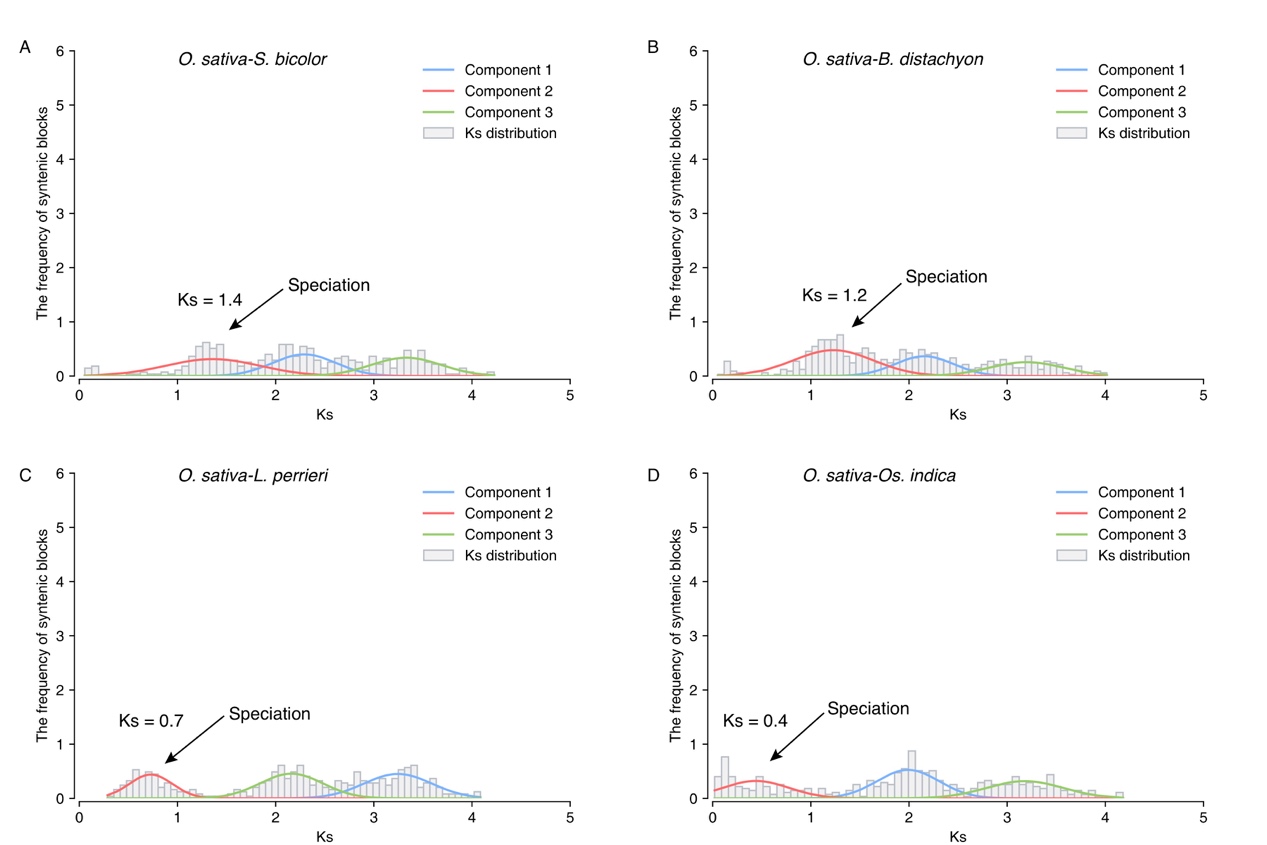


**Figure S18.** The estimation of time of speciation between *Oryza sativa* L. (ssp. japonica) lineage and *Sorghum bicolor*, *Brachypodium distachyon*, *Leersia perrieri* or *Oryza sativa* L. (ssp. indica) respectively. The Ks distribution was fitted using Gaussian mixture models (GMM).


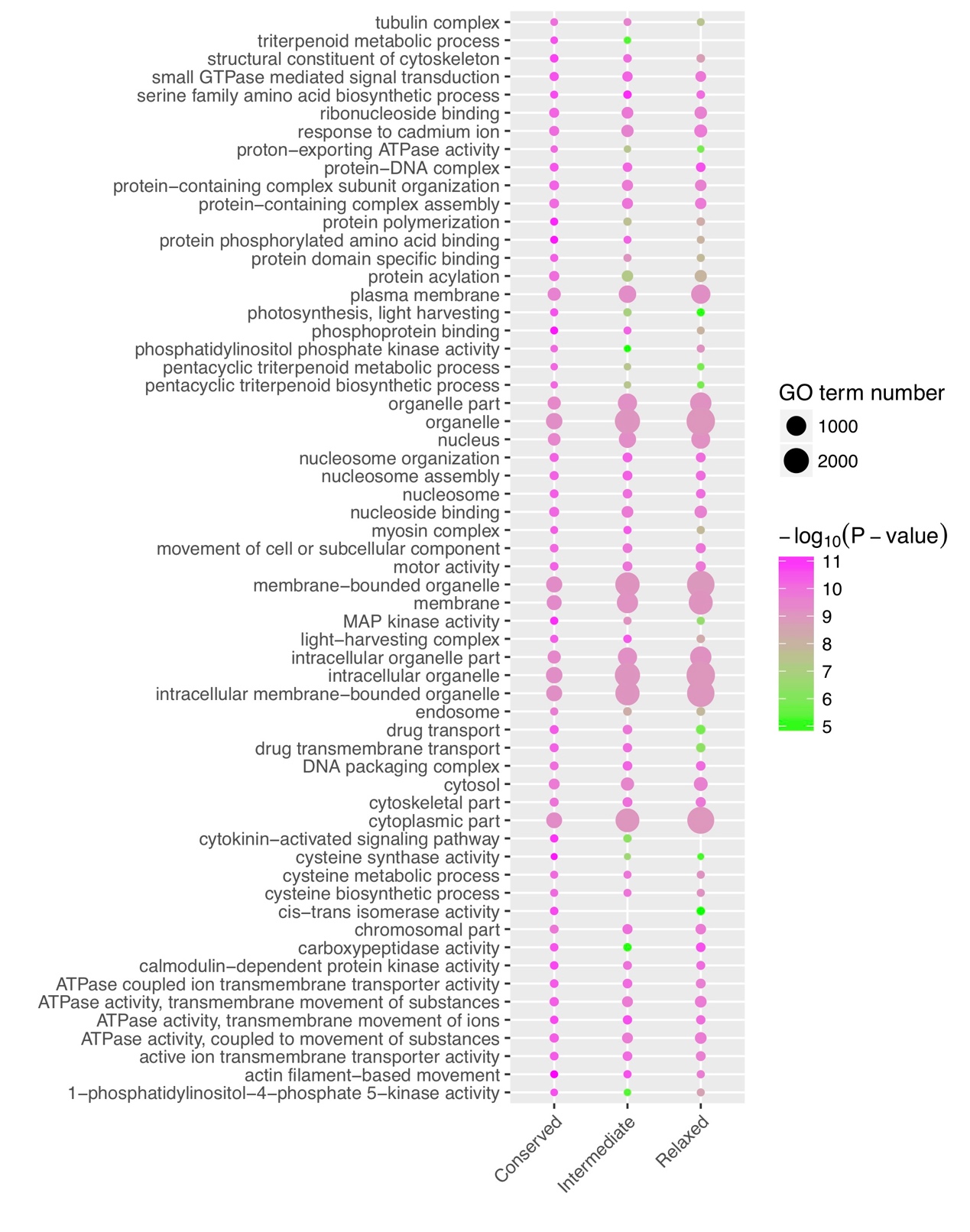


**Figure S19.** Functional enrichment analysis of most-preserved, intermediate-preserved and relaxed-preserved plant gene families in Arabidopsis. The enriched GO terms with corrected p-value < 0.01 are presented. The color of circle represents the statistical significance of enriched GO terms. The size of circle represents the number of a GO term.


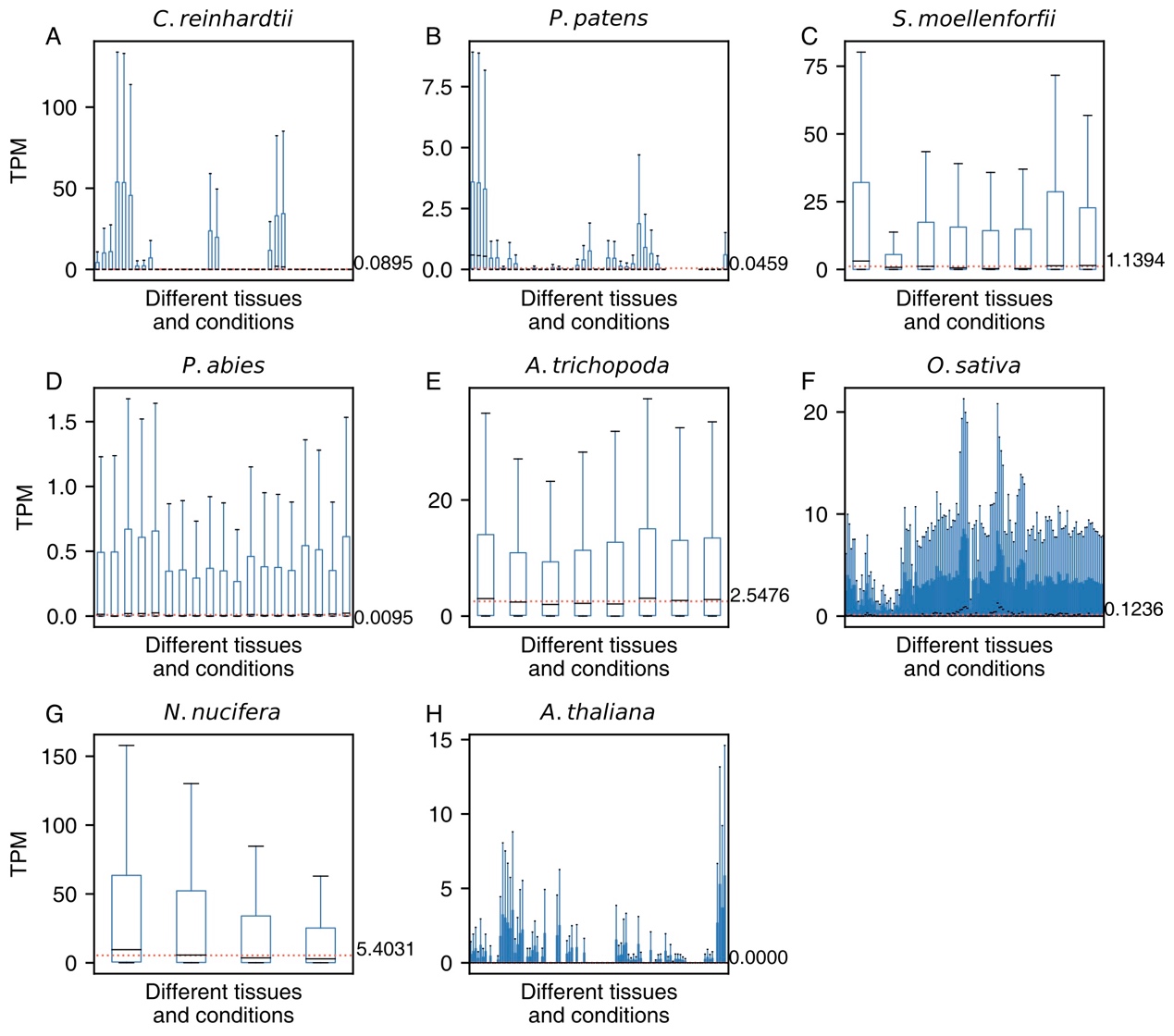


**Figure S20.** The distributions of TPM values in different tissues and conditions for intergenic sequences from different plants. The horizontal red line indicates the mean value of the medians in different boxplots in each subplot. (A) *Chlamydomonas reinhardtii*; (B) *Physcomitrella patens*; (C) *Selaginella moellendorffii*; (D) *Picea abies*; (E) *Amborella trichopoda*; (F) *Oryza sativa*; (G) *Nelumbo nucifera*; (H) *Arabidopsis thaliana*.


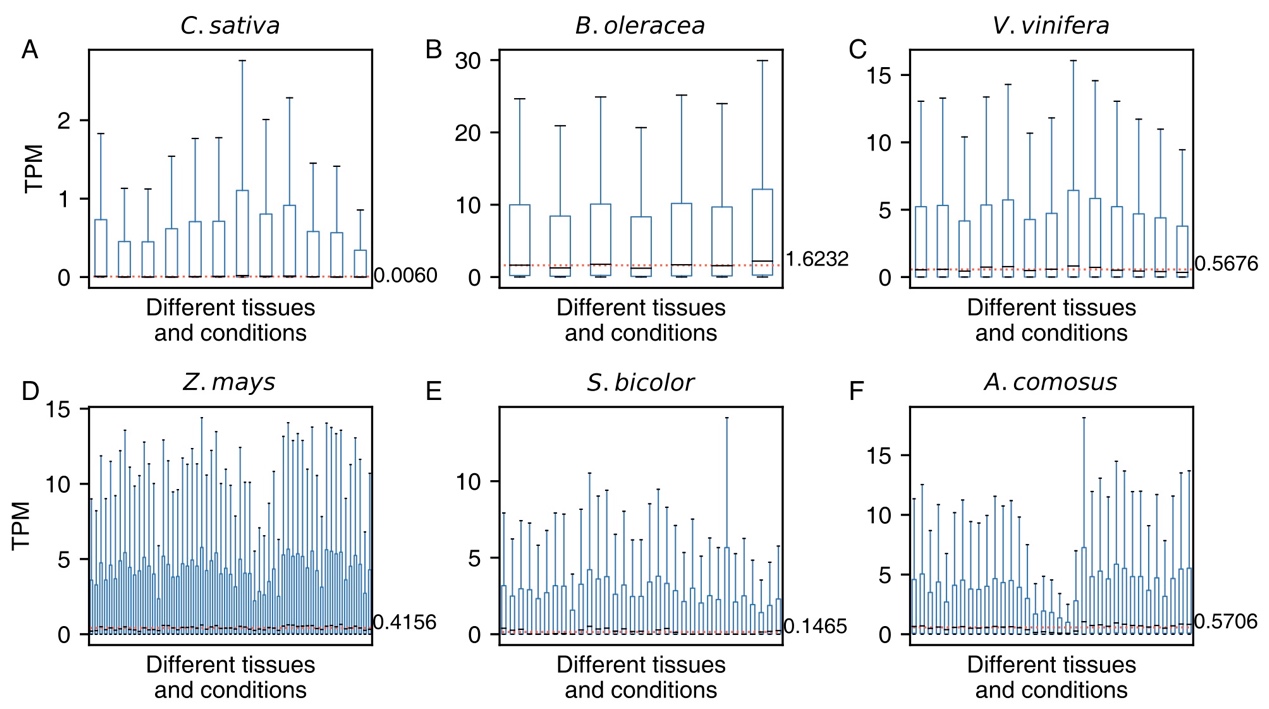


**Figure S21.** The distributions of TPM values in different tissues and conditions for intergenic sequences from different plants. The horizontal red line indicates the mean value of the medians in different boxplots in each subplot. (A) *Camelina sativa*; (B) *Brassica oleracea*; (C)*Vitis vinifera*; (D) *Zea mays*; (E) *Sorghum bicolor*; (F) *Ananas comosus*.
